# A novel AML1-ETO/FTO positive feedback loop promotes leukemogenesis and Ara-C resistance via stabilizing IGFBP2 in t(8;21) acute myeloid leukemia

**DOI:** 10.1186/s40164-024-00480-z

**Published:** 2024-01-24

**Authors:** Wei Zhou, Siying Li, Hong Wang, Jingfeng Zhou, Shuyi Li, Guofeng Chen, Wei Guan, Xianli Fu, Clara Nervi, Li Yu, Yonghui Li

**Affiliations:** 1 Central Laboratory, Shenzhen University General Hospital, Shenzhen University, Shenzhen, 518055 Guangdong China; 2 Guangdong Key Laboratory for Biomedical Measurements and Ultrasound Imaging, National-Regional Key Technology Engineering Laboratory for Medical Ultrasound, School of Biomedical Engineering, Shenzhen University Medical School, Shenzhen, 518060 Guangdong China; 3 School of Medicine, South China University of Technology, Guangzhou, 511400 Guangdong China; 4 Department of Hematology and Oncology, International Cancer Center, Shenzhen Key Laboratory of Precision Medicine for Hematological Malignancies, Shenzhen University General Hospital, Shenzhen University, Shenzhen, 518055 Guangdong China; 5 Department of Endoscopy, Tianjin Medical University Cancer Institute and Hospital, National Clinical Research Center for Cancer, Key Laboratory of Molecular Cancer Epidemiology of Tianjin, Key Laboratory of Cancer Prevention and Therapy, Tianjin’s Clinical Research Center for Cancer, Tianjin, 300060 China; 6 Senior Department of Hematology, The Fifth Medical Center of PLA General Hospital, Beijing, 100039 China; 7 Department of Pathology, Shenzhen University General Hospital, Shenzhen, 518055 Guangdong China; 8 Department of Medical and Surgical Sciences and Biotechnologies, University of Roma La Sapienza, 04100 Latina, Italy

**Keywords:** t(8;21) acute myeloid leukemia, AML1-ETO, N^6^-methyladenosine, FTO, Ara-C resistance

## Abstract

**Background:**

t(8;21)(q22;q22) is one of the most frequent chromosomal abnormalities in acute myeloid leukemia (AML), leading to the generation of the fusion protein AML1-ETO. Despite t(8;21) AML being considered as a subtype with a favorable prognosis, approximately 30–50% of patients experience drug resistance and subsequent relapse. N^6^-methyladenosine (m^6^A) is demonstrated to be involved in the development of AML. However, the regulatory mechanisms between AML1-ETO and m^6^A-related enzymes and the roles of dysregulated m^6^A modifications in the t(8;21)-leukemogenesis and chemoresistance remain elusive.

**Methods:**

Chromatin immunoprecipitation, dual-luciferase reporter assay, m^6^A-qPCR, RNA immunoprecipitation, and RNA stability assay were used to investigate a regulatory loop between AML1-ETO and FTO, an m^6^A demethylase. Gain- and loss-of-function experiments both in vitro and in vivo were further performed. Transcriptome-wide RNA sequencing and m^6^A sequencing were conducted to identify the potential targets of FTO.

**Results:**

Here we show that FTO is highly expressed in t(8;21) AML, especially in patients with primary refractory disease. The expression of FTO is positively correlated with AML1-ETO, which is attributed to a positive regulatory loop between the AML1-ETO and FTO. Mechanistically, AML1-ETO upregulates FTO expression through inhibiting the transcriptional repression of FTO mediated by PU.1. Meanwhile, FTO promotes the expression of AML1-ETO by inhibiting YTHDF2-mediated *AML1-ETO* mRNA decay. Inactivation of FTO significantly suppresses cell proliferation, promotes cell differentiation and renders resistant t(8;21) AML cells sensitive to Ara-C. FTO exerts functions by regulating its mRNA targets, especially IGFBP2, in an m^6^A-dependent manner. Regain of Ara-C tolerance is observed when IGFBP2 is overexpressed in FTO-knockdown t(8;21) AML cells.

**Conclusion:**

Our work reveals a therapeutic potential of targeting AML1-ETO/FTO/IGFBP2 minicircuitry in the treatment for t(8;21) patients with resistance to Ara-C.

**Supplementary Information:**

The online version contains supplementary material available at 10.1186/s40164-024-00480-z.

## Introduction

Acute myeloid leukemia (AML) is a highly heterogeneous disease characterized by complex cytogenetic and molecular landscape, leading to diverse prognostic outcomes [1, 2]. The t(8;21)(q22;q22) chromosomal translocation is one of the most common cytogenetic abnormalities in AML [3]. Clinically, t(8;21) AML has been categorized as a subtype with favorable prognosis; nonetheless, the outcomes of patients with this subtype still exhibit significant heterogeneity. Approximately 30–50% of t(8;21) AML patients experience relapse, resulting in poor clinical outcomes [4], suggesting the presence of genetic and clonal heterogeneity within this specific subtype of AML. Therefore, an in-depth understanding of the pathogenesis of t(8;21) AML will aid in improving personalized clinical interventions.

The AML1-ETO fusion protein, generated by the t(8;21)(q22;q22) chromosomal translocation, plays the pivotal role in the initiation of t(8;21) AML. AML1-ETO disturbs the normal biofunctions of core binding factors, blocks differentiation and induces aberrant self-renewal of hematopoietic stem cells, transforms them into preleukemic cells [5]. Cooperating gene mutations (including *c-KIT, FLT3* and* N/KRAS*, etc.) enhance the proliferative potential of the preleukemic cells as ‘second hit’ and eventually causes full-blown leukemia [3]. Additional pathological mechanisms, such as epigenetic regulation, work in collaboration to facilitate the development of t(8;21) AML [6, 7].

As the most abundant chemical modification in eukaryotic mRNAs, N^6^-methyladenosine (m^6^A) plays a crucial role in the initiation, progression, and drug resistance of human cancer [8–12]. Several studies have indicated that alterations in m^6^A modifications contribute to leukemia development: m^6^A writers (i.e., methyltransferase-like 3 (METTL3) [13, 14], methyltransferase-like 14 (METTL14) [15, 16] and Wilms’ tumor 1-associating protein (WTAP) [17]), erasers (i.e., fat mass and obesity-associated protein (FTO) [18, 19] and α-ketoglutarate-dependent dioxygenase AlkB homolog 5 (ALKBH5) [20, 21]), and readers (i.e., YTH domain family 2 (YTHDF2) [22, 23]) are found to act as oncogenes in AML. In particular, the m^6^A demethylase FTO is reported to be highly expressed in certain AML subtypes, including those carrying t(15;17)/PML-RARA, t(11q23)/MLL-rearrangements, *NPM1*, or *FLT3*-*ITD* mutations, and promote leukemogenesis by regulating its mRNA targets, especially *RARA* and *ASB2*, through m^6^A demethylation [18]. A recent study identifies that the high expression of FTO in *NPM1* mutant AML patients may be attributed to an intragenic long noncoding RNA within FTO. It is a result of intragenic transcription activation occurring in the final intron of FTO, induced by a non-random epigenetic event [24]. R-2-hydroxyglutarate, produced by mutant isocitrate dehydrogenase 1/2 (IDH1/2), inhibits the proliferation of leukemic cells by suppressing the demethylase activity of FTO and its downstream CEBPA/MYC-relevant pathway [19]. Moreover, elevated expression of FTO contributes to resistance to tyrosine kinase inhibitor in AML [25]. However, the definite regulatory relationship between FTO and leukemia-initiating fusion protein AML1-ETO and the role of FTO in progression and chemoresistance in t(8;21) AML remain largely unknown.

Here, we report a positive feedback regulatory loop between AML1-ETO and FTO: AML1-ETO promotes FTO expression via transcription factor PU.1; meanwhile, FTO stabilizes AML1-ETO mRNA via m^6^A reader YTHDF2, thus upregulating its expression. Suppression of FTO expression genetically or pharmacologically inhibits cell proliferation, promotes cell differentiation and resensitizes resistant t(8;21) AML cells to Cytosine arabinoside (Ara-C), via downregulating its functional downstream target IGFBP2. Our findings suggest a therapeutic potential of targeting AML1-ETO/FTO/IGFBP2 minicircuitry in the treatment of t(8;21) AML.

## Methods

### Samples from patients with leukemia and healthy donors

The collection of patient samples was approved by the Medical Ethics Committee of Shenzhen University General Hospitals. Mononuclear cell (MNC) isolated from bone marrow (BM) samples from patients with t(8;21) AML (Additional file 2: Table S1) and healthy donors were obtained with informed consent and stored in TRIzol reagent (Invitrogen) until use.

### Cell culture

The t(8;21) AML cell lines SKNO-1 and Kasumi-1 were maintained in RPMI-1640 medium (Gibco) supplemented with 10% fetal bovine serum (FBS, Gibco) and 1% penicillin–streptomycin (Solarbio). Human embryonic kidney (HEK)-293T cells were maintained in DMEM (Gibco) supplemented with 10% FBS and 1% penicillin–streptomycin.

### DNA site-directed mutagenesis

The pCMV6-wild type FTO-CDS (coding region sequence) plasmid was used to construct mutant FTO-CDS carrying H231A and D233A single-point mutations. The plasmid and mutation sites of FTO-CDS were kindly provided by Dr. Chuan He and Dr. Guifang Jia. DNA site-directed mutagenesis was performed using the Mut Express II Fast Mutagenesis Kit V2 (Vazyme), following the manufacturer’s instructions. Briefly, a pair of primers was designed based on the desired mutation sites. PCR amplification of target plasmids was performed using the designed primers. Amplification products were digested with DpnI, followed by homologous recombination reaction to cyclize the products. Cyclized products were used to transform competent bacterial cells, which were plated onto selective media overnight at 37 °C. Single bacterial colonies were identified by sequencing.

### Plasmid construction

Wild-type and mutant FTO-CDS were PCR-amplified from pCMV6-wtFTO and pCMV6-mutFTO, respectively, using the following primers: forward 5′-CTAGACTAGTATGAAGCGCACCCCGACTGCCGAGGAACGA-3′ and reverse 5′-ATTTGCGGCCGCCTAGGGTTTTGCTTCCAGAAGCTGA-3′, and then inserted into the pLVX lentiviral plasmid between SpeI and NotI. The DNA sequences encoding shRNAs targeting human FTO (shFTO#1 TRCN0000255405 and shFTO#2 TRCN0000255403), mouse Fto (TRCN0000277195, TRCN0000216341, and TRCN0000277143 mixed), human YTHDF2 (shYTHDF2#1 TRCN0000254336, shYTHDF2#2 TRCN0000254410), human IGFBP2 (shIGFBP2#1 TRCN0000006574 and shIGFBP2#2 TRCN0000006577), and mouse Igfbp2 (TRCN0000420942, TRCN0000422736, and TRCN0000012860 mixed) were commercially synthesized by TranSheep Bio Co. Ltd., and cloned into the pLKO.1 plasmid. Likewise, human PU.1-CDS and IGFBP2-CDS were commercially synthesized and cloned into the pTSB vector. For the experiments of silencing the three YTHDF proteins, the shRNA sequences targeting the three proteins were collectively inserted into a single pLKO.1 plasmid (DF1: TRCN0000294275; DF2: TRCN000254410; DF3: TRCN000365173 as #1 and DF1: TRCN0000286871; DF2: TRCN0000254336; DF3: TRCN0000167772 as #2).

### Lentiviral infection and siRNA transfection

Lentiviruses were generated from HEK-293T cells via transfection of lentiviral expression vectors together with plp-VSV-G:psPAX2 packaging mix (Addgene) using 15 μM PEI. Supernatants containing the virus were harvested 48 and 72 h after cotransfection and were added to target cells along with polybrene (8 μg/mL, Solarbio). Selection of infected cells was performed using puromycin (Solarbio, 1.5 μg/mL) 96 h after infection.

The siRNA targeting the fusion site of the AML1-ETO mRNA (siAE#1, sense, 5′-CCUCGAAAUCGUACUGAGAAG-3′, antisense, 5′-UCUCAGUACGAUUUCGAGGUU-3′; siAE#2, sense, 5′-CCUCGAAAUCGUACUGAGATT-3′, antisense, 5′-UCUCAGUACGAUUUCGAGGTT-3′) was designed according to previous studies [26] and was commercially synthesized by Sangon Biotech. siRNAs against human FTO (sc-75002), IGFBP2 (sc-37195), YTHDF2 (sc-78661) and YTHDF3 (sc-77724) were purchased from Santa Cruz Biotechnology. siRNAs were transfected into cells using the 4D-Nucleofector System (Lonza, Cologne, Germany) with the SF Cell Line 4D-Nucleofector X Kit by DS-138 for SKNO-1 and CM137 for Kasumi-1. Cells were harvested at 48 or 72 h after transfection.

The SKNO-1-siAE cell line was kindly provided by Dr. Clara Nervi which was generated by transfected with the lentiviral vector pRRLcPPT.hPGK encoding siAE oligonucleotides to silence the expression of AML1-ETO in SKNO-1 cells [6]. Kasumi-1-siAE cells were generated by siRNA transfection using the 4D-Nucleofector system.

### AML1-ETO9a-driven AML mice model

The animal experiments in our study were approved by the Ethics Committee of Shenzhen University General Hospital. BM and spleen cells isolated from AML1-ETO9a-driven AML mice were kindly provided by Dr. Lan Wang [27] and were further amplified by retransplantation into sublethally irradiated (450 cGy γ-rays) C57BL/6 recipient mice via tail vein injection (2 × 10^4^ cells/mouse). Freshly harvested BM and spleen cells were infected with pLKO.1-based lentiviral shRNA targeting mouse *Fto* or mouse *Igfbp2*, or pLKO.1-scrambled shRNA, and injected into sublethally irradiated (450 cGy) C57BL/6 recipient mice via tail vein injection (2 × 10^4^ cells/mouse). For FB23-2 treatment, FB23-2 was administered at 6 mg/kg daily for 30 days from the third day after transplantation.

### Xenograft model

Four- to six-week-old female BALB/c nude mice were used and maintained in a pathogen-free environment. After 3–5 days of adaptive feeding, each mouse received subcutaneous inoculation into the right flank with 2 × 10^7^ SKNO-1 cells in 200 µL of PBS. When the tumors reached a volume of approximately 150 mm^3^, the mice were randomly divided into 4 groups (n = 6 in each group). Then the mice in each group received intraperitoneal injection once a day of DMSO, Ara-C (75 mg/kg/day), FB23-2 (2 mg/kg/day), or a combination of both. The mice were sacrificed by cervical dislocation under anesthesia after two-week drug injection and the harvested xenograft tumors were analyzed further.

### NOD/SCID/IL2rγ^null^ immunodeficient mice model

The NOD/SCID/IL2rγ^null^ immunodeficient NSG mice (6–8 weeks old, female) were irradiated at 200 cGy and injected with GFP^+^ SKNO-1 cells (5 × 10^6^) via tail vein. The mice were randomly divided into 4 groups (n = 6 in each group), receiving intraperitoneal injection every other day of DMSO, Ara-C (75 mg/kg/day), FB23-2 (4 mg/kg/day), or a combination of both, starting one week after injection. The mice were sacrificed by cervical dislocation under anesthesia 6 weeks after AML cell injection.

### Cell proliferation and colony forming unit assays

The Cell Counting Kit-8 (Dojindo) was used to conduct cell proliferation assays. Cells were seeded at 30,000 cells/100 μL per well into 96-well plates in triplicate and incubated with 10 μL Cell Counting Kit-8 solution at 37 °C for 4 h. Absorbance was read using a microplate reader (Multiskan FC, Thermo Fisher) at 450 nm.

The MethoCult™ H4434 Classic (StemCell Technologies) culture system was used to perform colony-forming unit assays. Briefly, 1000 cells were mixed thoroughly with 4 mL MethoCult™ medium, seeded into 35 mm dishes in triplicate, and incubated for 14 days at 37 °C and 5% CO_2_ with humidification. Clusters that showed morphological hematopoietic characteristics containing more than 50 cells were counted as colonies.

### Flow cytometric analysis of apoptosis, cell cycle, and differentiation

The Annexin V-FITC Apoptosis Detection kit (Dojindo) was used for apoptosis assays. Cells were harvested and washed twice with cold PBS. After being suspended in 1 × Annexin V binding buffer, cells were stained with Annexin V-FITC and propidium iodide (PI) for 15 min on ice. For cell cycle assays, a DNA labeling solution (Cytognos) was used. Briefly, after collection and washing with cold PBS, cells were stained with DNA labeling solution for 15 min at 25 °C. For cell differentiation assays, human AML cells were harvested and washed with cold PBS; peripheral blood (PB), BM, and spleen cells were harvested from AML1-ETO9a AML mice, and erythrocytes were lysed using RBC Lysis Buffer (00-4333-57, Invitrogen). Human AML cells were stained with anti-human CD11b (301310, Biolegend) and isolated mice cells were stained with anti-CD11b-BV421 (101251, Biolegend) for 30 min on ice. Stained cells were analyzed using a CytoFLEX S Flow Cytometer (Beckman Coulter).

### RNA extraction and quantitative RT-PCR

Total RNA was extracted using TRIzol reagent (Invitrogen), in accordance with the manufacturer’s instructions. Briefly, 1 µg of total RNA was reverse-transcribed into cDNA using the GoScript™ Reverse Transcription System (Promega). Quantitative real-time PCR (qPCR) analysis was performed using 1 µL diluted cDNA (fivefold dilution) with the SYBR Fast Universal (KAPA Biosystems) on the ABI Prism 7500 sequence detection system (Applied Biosystems). Samples were run in triplicate, with GAPDH as the endogenous control. Additional file 2: Table S3 lists the primers used.

### Western blotting

Western blotting was performed with equal amounts of cell lysates (~ 20 μg), using the following antibodies: anti-FTO (#45980, 1:1000; Cell Signaling Technology), anti-AML1 (#4334, 1:1000; Cell Signaling Technology), anti-PU.1 (#2266, 1:1000; Cell Signaling Technology), anti-YTHDF2 (24744-1-AP, 1:1000; Proteintech), anti-YTHDF3 (sc-377119, 1:200; Santa Cruz Biotechnology), anti-IGFBP2 (#3922, 1:1000; Cell Signaling Technology), anti-Igfbp2 (sc-515134, 1:200; Santa Cruz Biotechnology), anti-β-actin hFAB rhodamine (12004163, 1:1000; Bio-Rad), and anti-β-actin (#4970, 1:1000; Cell Signaling Technology).

### m^6^A dot blot

Polyadenylated (poly(A)) mRNA was isolated from total RNA using the GenElute™ mRNA Miniprep Kit (Sigma-Aldrich). Isolated mRNA was denatured at 95 °C for 3 min and then dotted onto an Amersham Hybond™-N^+^ membrane (RPN203B, GE Healthcare) using a Bio-Dot Apparatus (#170-6545, Bio-Rad). After UV cross-linking, the membrane was blocked for 1 h using 5% nonfat milk, incubated at 4 °C with m^6^A antibody (ab232905, 1:400; Abcam) overnight, and then incubated with HRP-linked anti-rabbit immunoglobulin G (IgG, #7074, Cell Signaling Technology) for 1 h. Finally, the membrane was imaged using a ChemiDoc Touch Imaging System (Bio-Rad). For loading control, the RNA dotted membrane was stained with 0.02% methylene blue (Solarbio) diluted with 0.5M sodium acetate (PH 5.0).

### Chromatin immunoprecipitation (ChIP)

The SimpleChIP® Plus Enzymatic Chromatin IP Kit (#9005, Cell Signaling Technology) was used to conduct ChIP assays in accordance with the manufacturer’s instructions. Briefly, to crosslink proteins to DNA, cells were treated with formaldehyde (final concentration 1.5%) for 10 min. Micrococcal nuclease and sonication were used to digest chromatin to fragments of approximately 150–900 bp, which were then incubated with PU.1 antibody (#2266, Cell Signaling Technology), AML1 antibody (#sc-8563, Santa Cruz Biotechnology), ETO antibody (#sc-9737, Santa Cruz Biotechnology) or normal rabbit IgG antibody at 4 °C overnight with rotation. Finally, 1% of digested chromatin was preserved as an input sample and purified along with the immunoprecipitated complex. The primers using for ChIP-qPCR the listed in Additional file 2: Table S3.

### Dual-luciferase reporter assays

The DNA fragments of different lengths of the *FTO* promoter were amplified from human genomic cDNA by PCR using the primers listed in Additional file 2: Table S3 and inserted into the pGL3-basic vector (Promega) between KpnI and HindIII enzyme sites. The pcDNA3.1 vector containing DNA fragments of human PU.1-CDS were synthesized by Vigene Biosciences, Inc. Total 400 ng of pcDNA3.1 vector (i.e., pcDNA3.1-mock or pcDNA3.1-PU.1-CDS) and pGL3-basic vector (i.e., PGL3-P1 to PGL3-P4), and 20 ng pRL-TK Renilla luciferase reporter vector were cotransfected into HEK293T cells in 24-well plates using the SuperFect® Transfection Reagent (Qiagen). Luciferase activity was assessed 48 h after transfection using a Dual-Luciferase Reporter Assay System (Promega). Each experiment was repeated in triplicate.

The wild-type and m^6^A-mutant IGFBP2 3′ untranslated region (3′-UTR) were directly synthesized and inserted into the psiCHECK2 vector (Promega) by Vigene Biosciences, Inc. Briefly, 400 ng wild-type or mutant psiCHECK2-IGFBP2-3′-UTR and 100 ng *FTO*-expressing vector (i.e., pCMV6-wtFTO, pCMV6-mutFTO, or pCMV6-mock) were cotransfected into HEK-293T cells in 24-well plates with the SuperFect® Transfection Reagent (Qiagen). Relative luciferase activity was assessed using the Dual-Luciferase Reporter Assay System (Promega) 48 h posttransfection. Each experiment was repeated in triplicate.

### Gene-specific m^6^A-qPCR

The Magna MeRIP m^6^A Kit (17-10499, Millipore) was used to estimate the relative m^6^A levels in individual transcripts. Briefly, poly(A) mRNA was enriched from total RNA using the GenElute™ mRNA Miniprep Kit (Sigma-Aldrich), one-tenth of which was preserved as the input. Subsequently, mRNA was incubated overnight at 4 °C with protein A/G magnetic beads preconjugated with m^6^A antibody (MABE1006) or normal mouse IgG antibody (CS200621). The bead- immunoprecipitated methylated mRNAs were eluted by free m^6^A competition and purified using the RNeasy MiniElute Cleanup kit (#74204, Qiagen). Purified immunoprecipitated mRNAs and input sample were evaluated by qPCR using the primers listed in Additional file 2: Table S3.

### RNA immunoprecipitation (RIP) assay

The Magna RIP RNA-Binding Protein Immunoprecipitation Kit (17–700, Millipore) was used to perform RIP assays following the manufacturer’s instructions. Briefly, cell lysates were incubated at 4 °C overnight with magnetic beads preconjugated with antibodies against FTO (#31687, Cell Signaling Technology), YTHDF1 (#57530, Cell Signaling Technology), YTHDF2 (24744-1-AP, Proteintech), YTHDF3 (sc-377119, Santa Cruz Biotechnology), or mouse IgG. One-tenth of cell lysate was saved as an input sample. RNAs were eluted using proteinase K at 55 °C for 30 min. Eluted RNAs were purified using the RNeasy MiniElute Cleanup Kit (#74204 Qiagen). qPCR was performed to evaluate the interaction between YTHDF2 and mRNA transcripts. Primers used are listed in Additional file 2: Table S3.

### RNA stability

Cells were incubated with actinomycin D (final concentration 5 mg/mL) and harvested after 1, 1.5, or 3 h of incubation. Total RNA was extracted using the TRIzol reagent (Invitrogen), followed by qPCR. The mRNA degradation rate was estimated in accordance with previous studies [18] using the formula: In(C/C_0_) = − K_decay_t. The mRNA half-life of the decay t_1/2_ was calculated as follows: t_1/2_ = In2/K_decay._

### RNA-seq and m^6^A-seq

Wild-type *FTO*-overexpressing and control Kasumi-1 cell lines were used in triplicate to conduct RNA-seq (RNA sequencing) and m^6^A-seq (m^6^A sequencing). RNA-seq and m^6^A-seq were performed by LC-BIO Bio-tech Ltd. (Hangzhou, China). Briefly, total RNA was extracted from wild-type *FTO*-overexpressing (wtFTO) and control (mock) cells using the TRIzol reagent. The RNA integrity number (RIN) of total RNA was assessed using an Agilent Bioanalyzer 2100 with an RNA 6000 Nano LabChip Kit (Agilent) with RIN ≥ 7. Poly(A) mRNA was enriched from total RNA using poly-T oligo-attached magnetic beads (Invitrogen) and then fragmented using divalent cations at elevated temperatures. Fragmented mRNAs were incubated at 4 °C with m^6^A antibody (#202003, Synaptic Systems) for 2 h in IP buffer, and then incubated with protein-A beads for another 2 h at 4 °C. The mixture was eluted with elution buffer (6.7 mM m^6^A in IP buffer) and precipitated using 75% ethanol. The eluted IP and input mRNAs were used to construct a cDNA library with strand-specific library preparation using the dUTP method. Then 2 × 150 bp paired-end sequencing was performed on an Illumina Novaseq 6000 platform (Illumina). The accession number of these data in the Sequence Read Archive (SRA) database is PRJNA876028.

### RNA pull-down and mass spectrometry analysis

Biotin-labelled RNA oligonucleotides containing m^6^A or adenosine were directly synthesized by Sangon Biotech. The Pierce Magnetic RNA-Protein Pull-Down Kit (20164, Thermo Fisher Scientific) was used to perform RNA pull-down assays, following the manufacturer’s instructions. Briefly, up to 50 pmol of biotin-labelled RNAs was mixed with 50 μL streptavidin beads at 25 °C for 30 min and then incubated together with up to 200 μg of protein lysates with rotation for 1 h at 4 °C. After washing thrice, streptavidin beads were boiled, separated by 10% SDS-PAGE, and detected by western blot analysis or silver staining. Mass spectrometry analysis was performed by Qinglian Biotech Co. Ltd. Briefly, in-gel proteins were digested with trypsin. Peptides were then used for LC–MS/MS analysis on an EASY nLC 1200 ultra-high-pressure system (Thermo Fisher Scientific) with a nano-electrospray ion source attached to a quadrupole Orbitrap mass spectrometer (Q Exactive HF-X, Thermo Fisher Scientific).

The mass spectrometry proteomics data have been deposited to the ProteomeXchange Consortium via the iProX partner repository [28] with the dataset identifier PDX048441.

### Statistical analysis

Data are presented as the mean ± SD. The means between groups were compared using a two-tailed Student’s *t*-test. A *p* value < 0.05 was considered statistically significant. The estimated distribution of overall survival was evaluated using the Kaplan–Meier method and compared using the log-rank test.

## Results

### FTO is highly expressed in t(8;21) AML and positively correlated with AML1-ETO

**Fig. 1:**
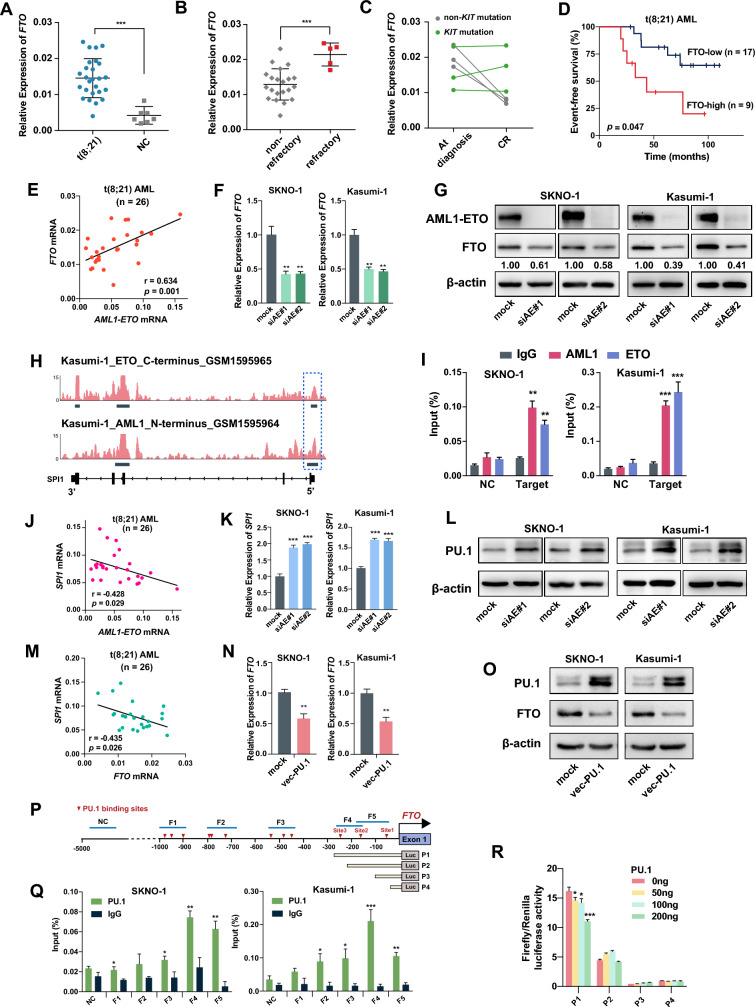
FTO is highly expressed in t(8;21) AML and upregulated by AML1-ETO via PU.1. **A** qPCR analysis of *FTO* mRNA expression in bone marrow (BM) cells from 26 patients with de novo t(8;21) AML and 7 normal controls (NC). **B** Comparison of *FTO* mRNA expression in BM cells from the 26 de novo t(8;21) patients with or without primary refractory AML using qPCR assay. **C** *FTO* mRNA expression in different stages of disease, including at diagnosis and achieving first CR, in the BM samples from 6 t(8;21) AML patients with or without *c-KIT* mutations by qPCR assay. **D** Comparison of event-free survival of patients with t(8;21) AML (n = 26) using the Kaplan–Meier method grouped by the levels of expression of FTO (high vs. low). **E** Pearson correlation of the *FTO* mRNA expression and *AML1-ETO* in BM cells from 26 patients with de novo t(8;21) AML. **F**, **G** Decreased FTO expression after knockdown of *AML1-ETO* (siAE) in SKNO-1 and Kasumi-1 cells detected by qPCR (**F**) and western blotting (**G**). **H** ChIP-seq of GSE65427 depicting the loci of *SPI1* in Kasumi-1 cells. The blue rectangle reveals a colocalization of AML1 (N-terminus) and ETO (C-terminus) targeting peaks, which represents AML1-ETO peaks, on the promoter of *SPI1* in Kasumi-1 cells. ChIP-seq peak regions were called using MACS at a *p* value cut-off of 10e−8 (Kasumi-1_ETO_C-terminus) or 10e−5 (Kasumi-1_AML1_N-terminus). **I** ChIP-qPCR assays showing the direct binding of AML1 and ETO within 200 bp upstream of the *SPI1* promoter in SKNO-1 and Kasumi-1 cells. The location of targeted amplified region (named ‘Target’) and negative control site (NC) are shown in detail in Additional file 1: Fig. S1D. **J** Pearson correlation of *SPI1* with the expression of *AML1-ETO* in BM cells from 26 patients with de novo t(8;21) AML. **K**, **L** qPCR (**K**) and western blotting (**L**) showing the increased expression of PU.1 after AML1-ETO knockdown (siAE) in SKNO-1 and Kasumi-1 cells. **M** Pearson correlation of *SPI1* with the expression of *FTO* in BM cells from 26 patients with de novo t(8;21) AML. **N**, **O** Decreased expression of FTO after forced expression of PU.1 in SKNO-1 and Kasumi-1 cells detected by qPCR (**N**) and western blotting (**O**). **P** Schematic diagrams of the PU.1 binding sites along the *FTO* promoter. Putative PU.1 binding sites were predicted by JASPER and are indicated with red vertical arrows. Blue horizontal lines indicate the amplified regions for ChIP-qPCR in **Q**. The lower panel indicates 4 *FTO* promoter fragments containing sites 1, 2, or 3 on the luciferase reporter vector. NC, negative control site. **Q** ChIP-qPCR assays showing the direct binding of PU.1 within 1 kb upstream of the *FTO* promoter in SKNO-1 and Kasumi-1 cells. The 5 qPCR amplified regions containing PU.1 putative binding sites predicted by JASPAR (S1–S5) and negative control site (NC) are shown in detail in **P**. **R** The effect of increasing amounts (50, 100, and 200 ng) of pcDNA3.0-PU.1-CDS on the relative luciferase activity of pGL3-basic vector containing different lengths of the *FTO* promoter (P1–P4, see **P** and Additional file 1: Fig. S1I), as analyzed by dual luciferase reporter assays

The expression levels of *FTO* were analyzed in BM cells collected from a cohort of patients with de novo t(8;21) AML (n = 26, Additional file 2: Table S1) and healthy donors (n = 7). Firstly, we found that the mRNA level of *FTO* in patients with de novo t(8;21) AML was significantly higher than that in healthy donors (Fig. 1A). Among these 26 patients with t(8;21) AML, 5 patients had at least one cycle of failed induction therapy and were classified as primary refractory t(8;21) AML (Additional file 2: Table S1). Interestingly, the data showed that the mRNA level of *FTO* was significantly higher in the patients with primary refractory disease compared with non-refractory patients (Fig. 1B). Furthermore, it is widely recognized that over 30% of t(8;21) AML patients harbor *c-KIT* mutations, which are strongly linked to a poor prognosis [29]. We found that t(8;21) AML patients without *c-KIT* mutation (n = 3, Pt# 1–3, Additional file 2: Table S1) exhibited a notable decrease in *FTO* mRNA levels upon achieving complete remission (CR); however, this phenomenon was not observed in patients with *c-KIT* mutation (n = 3, Pt# 4–6, Additional file 2: Table S1; Fig. 1C). We then grouped the 26 patients with de novo t(8;21) AML into tertiles according to the expression levels of *FTO*: FTO-high (n = 9) and FTO-low (n = 17) patients. Survival analysis revealed that FTO-high patients had significantly worse event-free survival (log-rank test, *p* = 0.047; Fig. 1D) and overall survival (log-rank test, *p* = 0.056; Additional file 1: Fig. S1A) than FTO-low patients with t(8;21) AML. Similar results were also observed in patients with AML (n = 344, GSE6891, Additional file 1: Fig. S1B). These data might suggest a potential role of FTO in the leukemia progression and chemotherapy resistance in t(8;21) AML.

To further explore the correlation between FTO and the t(8;21) AML-specific fusion protein AML1-ETO, we analyzed the expression of *AML1-ETO* and *FTO* in the 26 patients with de novo t(8;21) AML by qPCR. Interestingly, we found a significant positive correlation between the expression of *AML1-ETO* and *FTO* (Fig. 1E), suggesting a possible regulatory relationship between AML1-ETO and FTO.

### AML1-ETO upregulates expression of FTO via PU.1

AML1-ETO protein preserves the DNA binding capacity of AML1 and functions as a potent transcriptional repressor or activator through recruiting and forming oligomeric complexes with corepressors (NCoR/SMRT/DNMTs) or coactivators (PRMT1/p300) [30]. To investigate whether the expression of FTO is regulated by AML1-ETO, we silenced the expression of AML1-ETO in t(8;21) AML cell lines SKNO-1 and Kasumi-1. Western blotting and qPCR analysis indicated that silencing AML1-ETO led to reduced expression of FTO in t(8;21) AML cells (Fig. 1F, G). A similar result was observed in an RNA-seq dataset from GSE115121 in which the expression of *AML1-ETO* was knocked down in the Kasumi-1 cells [31] (Additional file 1: Fig. S1C). To further determine whether *FTO* is directly targeted by AML1-ETO, we analyzed a public chromatin immunoprecipitation sequencing (ChIP-seq) dataset that investigated the chromatin occupancy of AML1-ETO in the Kasumi-1 cells (GSE65427) [32]. However, the results showed that neither AML1 nor ETO protein might not have physical interaction with the *FTO* promoter (Additional file 1: Fig. S1D).

To identify other potential transcription factors that directly regulate the expression of FTO, we analyzed ENCODE ChIP-seq datasets using ChIPBase [33] and found that the hematopoietic transcription factor PU.1 (encoded by *SPI1* gene) bound directly to the *FTO* promoter within 1 kb upstream of the transcription start site (TSS) in the HL-60 cells, an acute promyelocytic leukemia cell line. Moreover, the ChIP-seq data from GSE65427 indicated that AML1-ETO could directly bind to the *SPI1* promoter (Fig. 1H). Consistent with ChIP-seq results of GSE65427, our results of ChIP-qPCR revealed the presence of AML1-ETO protein in the promoter region (− 183 to − 51 bp) of *SPI1* in SKNO-1 and Kasumi-1 cells (Fig. 1I and Additional file 1: Fig. S1E); negative results were showed when the expression of AML1-ETO was silenced (Additional file 1: Fig. S1F). Furthermore, AML1-ETO has been reported to inhibit the transcriptional activity of PU.1 by replacing the coactivator c-Jun from PU.1 [34]. Consistent with these findings, we found that the expression of *SPI1* was significantly negatively correlated with that of *AML1-ETO* in the BM samples from patients with t(8;21) AML (n = 26, Fig. 1J). Silencing AML1-ETO promoted the expression of PU.1 at both the mRNA and protein levels in t(8;21) AML cell lines (Fig. 1K, L). These findings suggest that the expression of PU.1 is transcriptionally repressed by AML1-ETO.

Furthermore, the expression of *SPI1* showed negative correlation with that of *FTO* in normal blood tissues from the Genotype-Tissue Expression Project (GTEx, n = 444, Additional file 1: Fig. S1G), BM samples from patients with AML from the Analysis of the Cancer Genome Atlas (TCGA) database (n = 173, Additional file 1: Fig. S1H), and BM samples from patients with t(8;21) AML (n = 26, Fig. 1M). Overexpression of PU.1 significantly decreased the expression of FTO in t(8;21) AML cells (Fig. 1N, O). Using JASPAR, we predicted 12 putative binding sites of PU.1 within 1 kb upstream of the TSS of *FTO*, according to which the *FTO* promoter was divided into 5 fragments (~ 100 to 150 bp in length, named F1–F5) for ChIP-qPCR amplification (Fig. 1P). ChIP-qPCR assays indicated that PU.1 was enriched on F4 and F5 at levels approximately fourfold greater than the immunoglobulin G (IgG) control in SKNO-1 and Kasumi-1 cells (Fig. 1Q), suggesting a direct interaction between PU.1 protein and the *FTO* promoter. Sites 1–3 (located in F4 and F5, see Fig. 1P) might be PU.1 binding sites on the *FTO* promoter. We then constructed reporter vectors containing different lengths of the *FTO* promoter sequences covering sites 1, 2, and 3 (including sequences P1 to P4 showed in Fig. 1P and Additional file 1: Fig. S1I, which were truncated sequentially according to the location of sites 1, 2, and 3 in Fig. 1P). We observed that compared with the empty vector, the reporter vector bearing the P1 sequence showed a dose-dependent repression of luciferase activity when cotransfected with an increasing dose of the PU.1-expressing vector, whereas repression was not observed for the vectors bearing P2 to P4 (all of which did not contain site 3), suggesting that site 3 might be the functional PU.1 binding site (Fig. 1R). This finding also supported the PU.1-dependent negative regulation of FTO expression. Together, these data suggest that AML1-ETO upregulates the expression of FTO via inhibiting the PU.1-induced transcriptional repression of FTO in t(8;21) AML cells.

### Expression of AML1-ETO is upregulated by FTO in an m^6^A-dependent manner mediated by YTHDF2

**Fig. 2:**
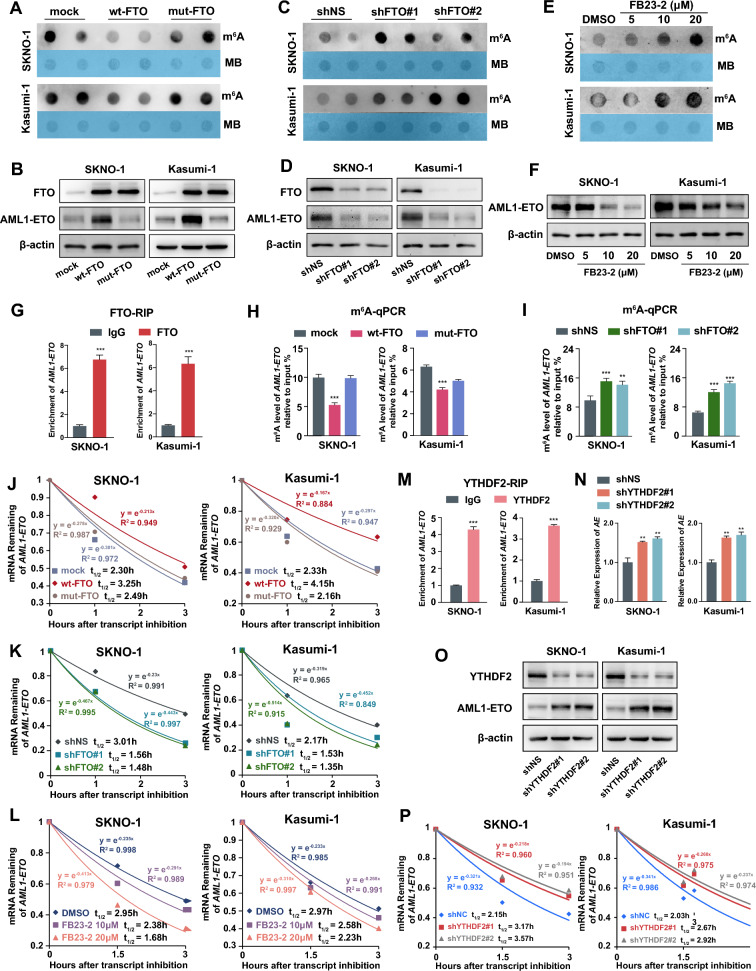
FTO promotes expression of AML1-ETO as an m^6^A demethylase by repressing YTHDF2-mediated *AML1-ETO* mRNA decay. **A**, **B** The m^6^A abundance in mRNA detected by m^6^A dot blotting (**A**) and the expression levels of FTO and AML1-ETO by western blotting (**B**) in SKNO-1 or Kasumi-1 AML cells with forced expression of wild-type FTO (wt-FTO), mutant FTO (mut-FTO), or mock vectors; Methylene blue (MB) represents the loading control of RNA samples. **C**, **D** The m^6^A dot blotting (**C**) and western blotting (**D**) in SKNO-1 or Kasumi-1 AML cells transfected with *FTO* shRNA or scramble shRNA (shNS) vectors. **E**, **F** Effects of FB23-2 treatment for 72 h on the m^6^A abundance in mRNA by m^6^A dot blotting (**E**) and expression levels of AML1-ETO detected by western blotting (**F**) in SKNO-1 and Kasumi-1 cells. **G** RIP-qPCR showing the interaction between *AML1-ETO* mRNA transcripts and FTO protein in SKNO-1 and Kasumi-1 cells. **H**, **I** The levels of m^6^A in AML1-ETO transcripts assessed by gene-specific m^6^A qPCR assay in SKNO-1 and Kasumi-1 cells transduced with wt-FTO, mut-FTO, or mock vector (**H**); or with *FTO* shRNA or shNS vectors (**I**). **J**–**L** The mRNA half-life (t_1/2_) of *AML1-ETO* transcripts in SKNO-1 and Kasumi-1cells transduced with wt-FTO, mut-FTO, or mock vector (**J**); or with *FTO* shRNA or shNS vectors (**K**) or treated with DMSO or FB23-2 for 72 h (**L**). **M** RIP-qPCR showing the interaction between *AML1-ETO* mRNA transcripts and YTHDF2 protein in SKNO-1 and Kasumi-1 cells. **N**, **O** Increased expression of AML1-ETO after YTHDF2 knockdown assessed by qPCR (**N**) and western blotting (**O**) in SKNO-1 and Kasumi-1 cells. **P** Increased mRNA half-life (t_1/2_) of AML1-ETO transcripts in SKNO-1 and Kasumi-1 cells after YTHDF2 knockdown

Given the significant positive correlation observed between the expression levels of *AML1-ETO* and *FTO* in patients, and the role of FTO as an m^6^A demethylase, we were curious to investigate whether FTO could also promote the expression of AML1-ETO. We found that in both SKNO-1 and Kasumi-1 cells, overexpression of wild-type FTO, but not mutant FTO (2 point mutations, H231A and D233A that disrupt the enzymatic activity of FTO [18]), substantially repressed the global mRNA m^6^A levels (Fig. 2A) and upregulated the expression of AML1-ETO at both the mRNA and protein levels (Additional file 1: Fig. S2A and Fig. 2B). Conversely, knocking down the endogenous expression of FTO by short hairpin RNA (shRNA) noticeably increased global mRNA m^6^A levels and reduced the expression of AML1-ETO (Fig. 2C, D; Additional file 1: Fig. S2B). Moreover, treatment with FB23-2, an FTO inhibitor that directly targets FTO and selectively inhibits its m^6^A demethylase activity, markedly decreased the expression of AML1-ETO in a dose-dependent manner (Fig. 2E, F; Additional file 1: Fig. S2C). RIP assay demonstrated the direct binding of FTO to *AML1-ETO* transcripts (Fig. 2G). To further confirm the role of FTO-mediated m^6^A demethylation in regulating the expression of AML1-ETO, we performed gene-specific m^6^A qPCR assays, and found that the levels of m^6^A modification on *AML1-ETO* mRNA transcripts were markedly decreased when wild-type FTO, but not its mutant, was overexpressed (Fig. 2H). Knocking down the endogenous expression of FTO resulted in opposite outcomes (Fig. 2I). These data indicate that FTO enhances the expression of AML1-ETO through decreasing the m^6^A level of *AML1-ETO* mRNA.

The m^6^A modification is known to regulate the stability of mRNA [35]. To explore whether FTO modulates the expression of AML1-ETO by affecting its mRNA stability, we performed RNA stability assays and demonstrated that overexpression and knockdown of FTO prolonged and shortened the half-life of *AML1-ETO* mRNA transcripts, respectively (Fig. 2J, K). Similarly, when t(8;21) AML cells were treated with FB23-2, the half-life of *AML1-ETO* mRNA transcripts were noticeably decreased (Fig. 2L). It suggests that the upregulation of the expression of AML1-ETO is at least partially due to the increased stability of *AML1-ETO* transcripts mediated by FTO-induced m^6^A demethylation.

Previous studies have identified that the m^6^A reader YTHDF2 is responsible for recognizing m^6^A-modified mRNAs, locating them to the RNA decay site, and transforming them from the translation to the degradation state [35, 36]. To elucidate whether YTHDF2 is the m^6^A reader of *AML1-ETO*, we performed RIP assays and found that YTHDF2 bound to *AML1-ETO* mRNA directly (Fig. 2M). Accordingly, we detected that YTHDF2 knockdown by shRNA promoted the expression of AML1-ETO at both the mRNA and protein levels (Fig. 2N, O). Furthermore, RNA stability assays demonstrated a prolonged half-life of *AML1-ETO* mRNA transcripts when the expression of YTHDF2 was knocked down (Fig. 2P). It suggests that YTHDF2 may be one of the m^6^A readers that target and regulate the stability of *AML1-ETO* mRNA.

### FTO is essential in enhancing leukemogenesis of t(8;21) AML cells in vitro and in vivo

**Fig. 3:**
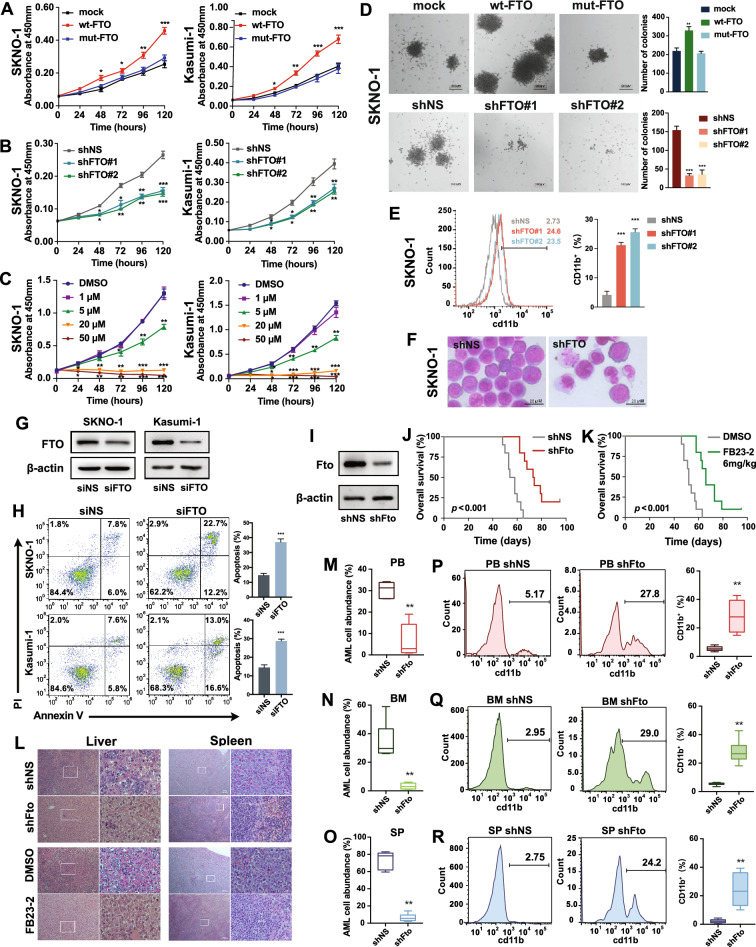
Biological impact of FTO in t(8;21) AML cells and AML1-ETO9a mice model. **A**–**C** Effects of forced expression of wild-type FTO (wt-FTO), mutant FTO (mut-FTO) or mock vectors (**A**); or FTO shRNA or scramble vectors (**B**), or FB23-2 treatment (**C**) on the proliferation of SKNO-1 and Kasumi-1 cells by CCK-8 assays. shNS: scramble shRNA. **D** Effects of forced expression or knockdown of FTO on colony-forming capacity of SKNO-1 cells. **E** The effect of FTO knockdown on differentiation of SKNO-1 cells. The percentage of CD11b^+^ cells was quantified (right panel). **F** Wright-Giemsa staining of SKNO-1 cells with or without FTO knockdown. **G**, **H** Effect of silencing the expression of FTO, confirmed by western blotting (**G**), on cell apoptosis in SKNO-1 and Kasumi-1 cells 72 h after siRNA transfection (**H**). **I** Western blotting of the *Fto* knockdown in AML1-ETO9a-driven AML mice cells. **J**, **K** Kaplan–Meier survival curves of AML1-ETO9a-driven AML mice (*n* = 10 for each group) with or without *Fto* knockdown (**J**) or treatment with DMSO or FB23-2 (**K**). **L** Hematoxylin and eosin (H&E) staining of liver (left panel) and spleen (right panel) of AML1-ETO9a-driven AML mice 7 weeks after transplantation. **M**–**O** Percentage of GFP^+^ AML1-ETO9a AML cells in the **M** peripheral blood (PB), **N** bone marrow (BM), and **O** spleen (SP) of the mice with or without *Fto* knockdown assessed by flow cytometric analysis. **P**–**R** Flow cytometric analysis of the distribution of anti-CD11b-stained GFP + AML1-ETO9a AML cells in PB (**P**), BM (**Q**), and SP (**R**) of mice with or without *Fto* knockdown

To elucidate whether FTO is crucial for the development and progression of t(8;21) AML, we performed gain- and loss-of-function experiments in vitro and vivo. Forced expression of wild-type but not mutant FTO significantly enhanced cell proliferation, increased the colony-forming capacity and promoted the transition of cells from G1 to S phase of the cell cycle in the two t(8;21) AML cell lines; conversely, when the expression or activity of FTO was inhibited by shRNA or the FTO inhibitor FB23-2, respectively, the opposite phenomena were observed (Fig. 3A–D and Additional file 1: Fig. S3A–B). Flow cytometry and Wright-Giemsa staining showed that the FTO knockdown promoted differentiation of t(8;21) AML cells (Fig. 3E–F; Additional file 1: Fig. S3C–D). Moreover, silencing FTO significantly promoted the apoptosis of t(8;21) AML cells (Fig. 3G, H). Next, we used an AML1-ETO9a-driven leukemic mice model to confirm the functional importance of FTO in vivo. The results showed that *Fto* knockdown (Fig. 3I) and injection of FB23-2 significantly delayed AML1-ETO9a-induced leukemogenesis in recipient mice (log-rank test, *p* < 0.001 and *p* < 0.001, respectively; Fig. 3J, K). Inhibition of the expression of *Fto* by shRNA and FB23-2 both noticeably suppressed splenomegaly caused by leukemic infiltration (Additional file 1: Fig. S3E–F). Wright-Giemsa staining and Hematoxylin and eosin (H&E) staining also demonstrated less leukemic cell dissemination in the bone marrow (Additional file 1: Fig. S3G), liver and spleen (Fig. 3L) of *Fto*-knockdown and FB23-2-treated mice. As determined by flow cytometric analysis, the abundance of leukemic cells from peripheral blood (PB), BM, and spleen samples was significantly suppressed in recipient mice when the expression of *Fto* was inhibited (Fig. 3M–O and Additional file 1: Fig. S3H–J). In addition, we detected that the proportion of CD11b-stained leukemic cells from PB, BM, and spleen samples was significantly increased in *Fto*-knockdown and FB23-2-treated recipient mice compared with that in the vehicle control (Fig. 3P–R and Additional file 1: Fig. S3K–M), suggesting that inhibition of FTO promoted the differentiation of t(8;21) AML cells in vivo. In summary, these data indicate the essential oncogenic role of FTO in t(8;21) leukemogenesis.

### FTO is crucial for the sensitivity of t(8;21) AML cells to Ara-C

**Fig. 4:**
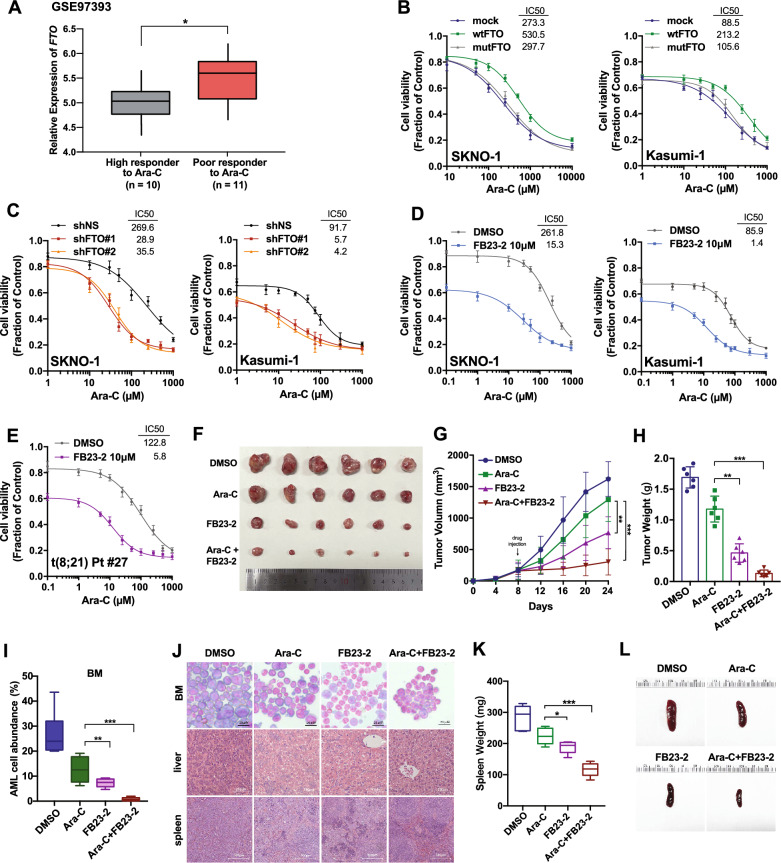
Suppression of FTO resensitizes resistant cells to Ara-C in vitro and in vivo. **A** Comparison of *FTO* mRNA expression in BM samples from AML patient with high versus poor response to Ara-C (GSE97393). **B**, **C** CCK-8 assays for SKNO-1 and Kasumi-1 cells transfected with wild-type FTO (wt-FTO), mutant FTO (mut-FTO) or mock vectors (**B**); or FTO shRNA or scramble vectors (**C**) treated with varying concentrations of Ara-C for 48 h. **D** CCK-8 assays for SKNO-1 and Kasumi-1 cells treated with 10 µM FB23-2 for 6 h followed by co-treatment of different concentrations of Ara-c for 48 h. **E** CCK-8 assays for primary BM cells collected from a relapsed t(8;21) AML patients with 10 µM FB23-2 treatment for 6 h followed by co-treatment of different concentrations of Ara-c for 48 h. **F** The external view of nude mice bearing SKNO-1 cell xenografts (n = 6 for each group) treated with DMSO, Ara-C, FB23-2, or a combination of Ara-C and FB23-2. **G**, **H** The growth curve of tumor volume (**G**) and the final tumor weight (**H**) for each group as indicated in **F**. **I**–**L** NOD/SCID/IL2rγ^null^ immunodeficient NSG mice injected with SKNO-1 cells through tail vein treated with DMSO, Ara-C, FB23-2, or a combination of Ara-C and FB23-2 (n = 6 for each group). **I** Blast cells percentage in bone marrow (BM), **J** Wright-Giemsa staining of bone marrow and H&E staining of livers and spleens, **K** spleen weights and **L** representative external views of the spleens were shown

Chemotherapy resistance is a leading cause of refractory or relapse AML. Ara-C is the core component of the standard "7+3" induction therapy of AML [37]. For t(8;21) AML, 2–4 cycles of high-dose Ara-c have been applied as the main therapeutic regimen for post-remission consolidation treatment. A transcriptome sequencing of primary BM cells harvested from AML patients at diagnosis (GSE97393) [38] showed that mRNA expression levels of FTO were significantly higher in patients with poor response to Ara-C (n = 11) in vivo compared with high responders (n = 10; Fig. 4A), suggesting a potential role of FTO in Ara-C resistance. We further investigated whether FTO impacts the sensitivity of t(8;21) AML cells to Ara-C in t(8;21) AML cells. Both SKNO-1 and Kasumi-1 are multi-drug resistant AML cell lines, established from relapsed t(8;21) AML patients who were resistant to chemotherapy [39, 40] and they were used as Ara-C resistant cell models. As assessed by proliferation assays, FTO overexpression and knockdown respectively exacerbated and restored the sensitivity to Ara-C in the two Ara-C resistant cell lines (Fig. 4B, C). Moreover, the SKNO-1 and Kasumi-1 cells that pre-exposed to 10 µM FB23-2 for 6 h to suppress FTO activity and followed by Ara-C treatment for additional 48 h were found to have notably lower IC_50_ values (Fig. 4D) and higher rates of apoptosis (Additional file 1: Fig. S4A and S4B). We observed similar results of proliferation assay in primary cells isolated from bone marrow of a relapse patient with t(8;21) AML (Pt #27, Additional file 2: Table S1) when they were treated with FB23-2 and Ara-C (Fig. 4E).

Nude mice engrafted with SKNO-1 cells was utilized to examine these effects in vivo. The combination therapy of Ara-C with FB23-2 successfully inhibited the tumor growth (Fig. 4F), with the smallest tumor volume (DMSO, 1620 ± 301 mm^3^; Ara-C, 1294 ± 346 mm^3^; FB23-2, 767 ± 253 mm^3^; Ara-C plus FB23-2, 304 ± 193 mm^3^; Fig. 4G) and lowest tumor weight (DMSO, 1.69 ± 0.17 g; Ara-C, 1.21 ± 0.21 g; FB23-2, 0.46 ± 0.15 g; Ara-C plus FB23-2, 0.13 ± 0.05 g; Fig. 4H) compared with administration of Ara-C or FB23-2 alone. Orthotopic xenograft using SKNO-1 cells was further performed in NOD/SCID/IL2rγ^null^ immunodeficient NSG mice. The combination therapy of Ara-C with FB23-2 dramatically inhibited the infiltration of leukemic cells in PB (Additional file 1: Fig. S4C), BM (Fig. 4I, J), liver (Fig. 4J) and spleen (Fig. 4J–L and Additional file 1: Fig. S4D) compared with administration of Ara-C or FB23-2 alone. These data support the potential of FTO inhibition in enhancing the sensitivity of t(8;21) AML cells to Ara-C.

### Identification of potential targets of FTO in t(8;21) AML by m^6^A-seq and RNA-seq

**Fig. 5:**
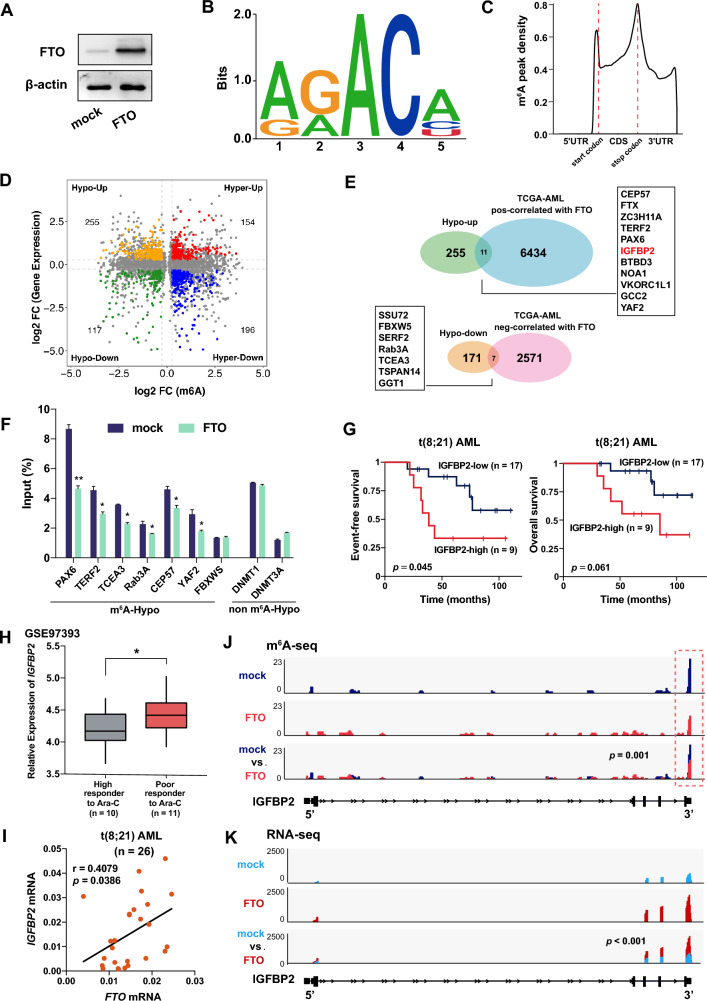
Transcriptome-wide identification of FTO targets in t(8;21) AML. **A** Western blot analysis of the expression of FTO in Kasumi-1 cells overexpressing or not wild-type FTO. **B** The predominant consensus motif RRACH (R[G/A]; H[U/A/C]) analyzed by HOMER in m^6^A-seq (*p* = 1e−10823). **C** Density distribution of m^6^A peaks across the length of mRNA transcripts. Each region of 5′-UTR, coding region (CDS), and 3′-UTR was split into 100 segments, and the percentage of m^6^A peaks that fall within each segment was determined. **D** Distribution of peaks with a significant change in both the m^6^A and RNA levels in *FTO*-overexpressing Kasumi-1 cells compared with control Kasumi-1 cells. **E** Venn diagram for the overlapping genes enriched in m^6^A hypomethylated genes in m^6^A-seq and FTO-expression-correlated genes in TCGA-AML database. **F** Confirmation of changes in the levels of m^6^A of 7 representative m^6^A-hypo genes and 2 non-m^6^A-hypo genes in Kasumi-1 cells by gene-specific m^6^A qPCR. **G** Kaplan–Meier analysis of event-free survival (left) and overall survival (right) of patients with de novo t(8;21) AML (n = 26) based on the expression of *IGFBP2*. **H** Comparison of *IGFBP2* mRNA expression in BM samples from AML patient with high versus poor response to Ara-C (GSE97393). **I** Pearson correlation of the expression of *IGFBP2* and *FTO* in BM cells from 26 patients with de novo t(8;21) AML. **J** The m^6^A level in *IGFBP2* mRNA transcripts in *FTO*-overexpressing and control Kasumi-1 cells as detected by m^6^A-seq. The m^6^A peaks shown in red rectangles reveal a significant decrease in abundance in *FTO*-overexpressing compared with mock cells (fold change = 0.72, *p* = 0.001). The abundance of m^6^A peaks after input normalization are shown. **K** The expression level of *IGFBP2* mRNA in *FTO*-overexpressing and control Kasumi-1 cells as detected by RNA-seq (fold change = 2.22, *p* < 0.001)

To investigate the potential targets of FTO in t(8;21) AML cells, we conducted m^6^A-seq and RNA-seq assays using wild-type *FTO*-overexpressing and control Kasumi-1 cells (Fig. 5A). As shown in Fig. 5B, a significant enrichment of the m^6^A motif RRACH was observed in m^6^A peaks. Moreover, the m^6^A peaks were largely enriched in exons (~ 92%; Additional file 1: Fig. S5A), especially surrounding the stop codon (Fig. 5C and Additional file 1: Fig. S5B).

Considering the m^6^A "eraser" role of FTO, we only regarded the mRNA transcripts that carried m^6^A peaks with decreasing abundance, namely m^6^A hypomethylated peaks, upon FTO overexpression as potential targets of FTO. Combined with RNA-seq data, we found 372 hypomethylated peaks in *FTO*-overexpressing cells relative to control cells, among which 255 mRNA transcripts were significantly upregulated (hypo-up, fold change ≥ 1.2;* p* < 0.05), whereas 117 were downregulated (hypo-down, fold change ≤ 0.83; *p* < 0.05, Fig. 5D), suggesting that the majority of m^6^A hypomethylated mRNA transcripts (68.5%) were associated with upregulated mRNA levels in *FTO*-overexpressing cells. GSEA analysis [41] indicated that the hypo-up transcripts were significantly enriched with target genes of SOX2 and NANOG, BRCA1 and CHEK2 network, and many important biological processes, such as cell cycle, immune system and RNA metabolism (Additional file 1: Figure S5C). Hypo-down transcripts were significantly enriched with genes involving the BRCA1 network (Additional file 1: Figure S5D).

We further analyzed the expression correlations between *FTO* and the m^6^A hypomethylated genes in AML patient samples from the TCGA dataset. We found that 11 hypo-up (*CEP57, FTX, ZC3H11A, TERF2, PAX6, IGFBP2, BTBD3, NOA1, VKORC1L1, GCC2,* and *YAF2*) and 7 hypo-down (*SSU72, FBXW5, SERF2, Rab3A, TCEA3, TSPAN14,* and *GGT1*) genes demonstrated a significantly positive and negative correlation in expression, respectively, with *FTO* in TCGA AML datasets (Fig. 5E and Additional file 2: Table S2). In agreement, gene-specific m^6^A qPCR assays confirmed that among the 7 m^6^A-hypo genes (*PAX6, TERF2, TCEA3, Rab3A, CEP57, YAF2,* and *FBXWS*), 6 were associated with a significant reduction in the m^6^A levels (86%), whereas 2 non-m^6^A-hypo genes (*DNMT1* and *DNMT3A*) in m^6^A-seq data did not demonstrate a significant decrease in m^6^A abundance (Fig. 5F). This verifies the reliability of our m^6^A-seq data.

### IGFBP2 is directly targeted by FTO via its m^6^A demethylase activity

We further found that the high expression of *IGFBP2* (insulin-like growth factor binding protein 2), which was listed in Fig. 5E, was significantly upregulated in patients with t(8;21) AML (n = 30) compared with normal control (n = 9, GSE30285 and GSE34814, Additional file 1: Fig. S5E). Moreover, IGFBP2 was associated with significantly worse event-free survival and overall survival in patients with AML (GSE6891, n = 344, log-rank test, *p* < 0.001 and *p* = 0.006, respectively; Additional file 1: Fig. S5F) and t(8;21) AML (n = 26, log-rank test, *p* = 0.045 and *p* = 0.061, respectively; Fig. 5G). The primary BM cells from AML patients at diagnosis with poor response to Ara-C had a significantly higher level of IGFBP2 mRNA compared with high responders (GSE97393; Fig. 5H). Moreover, IGFBP2 has been shown to support the migration and survival of AML cells [42]. We observed that *IGFBP2* manifested a significant positive association with the expression of *FTO* in AML cohorts from the TCGA database (Additional file 1: Fig. S5G) and our in-house t(8;21) AML cohorts (Fig. 5I). Our m^6^A-seq and RNA-seq data indicated that overexpression of FTO caused a significant reduction of m^6^A levels in the 3′ untranslated region (3′-UTR) of IGFBP2 mRNA (fold change = 0.72, *p* = 0.001, Fig. 5J) and significant increase level of IGFBP2 transcripts (fold change = 2.22, *p* < 0.001; Fig. 5K). We thus aimed at IGFBP2 as a potential target of FTO for further investigation.

**Fig. 6:**
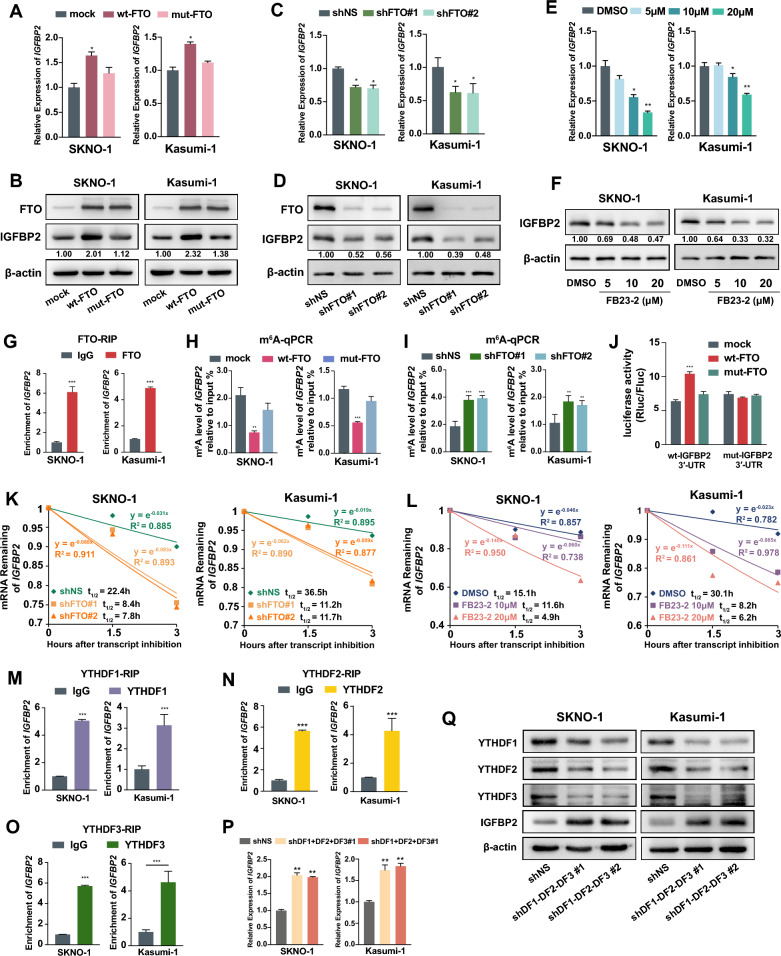
FTO promotes the expression of IGFBP2 in an m^6^A-dependent manner. **A**, **B** Expression of IGFBP2 assessed by qPCR (**A**) and western blotting (**B**) in SKNO-1 or Kasumi-1 cells transduced lentivirally with wild-type FTO (wt-FTO), mutant FTO (mut-FTO), or mock vectors. **C**, **D** Decreased expression of IGFBP2 detected by qPCR (**C**) and western blotting (**D**) in SKNO-1 and Kasumi-1 cells after knockdown of *FTO* by shRNAs. **E**, **F** Expression of IGFBP2 analyzed by qPCR (**E**) and western blotting (**F**) in SKNO-1 or Kasumi-1 cells after FB23-2 treatment for 72 h. **G** RIP-qPCR showing the interaction between *IGFBP2* mRNA transcripts and FTO protein in SKNO-1 and Kasumi-1 cells. **H**, **I** The levels of m^6^A in *IGFBP2* transcripts assessed by gene-specific m^6^A qPCR assay in SKNO-1 and Kasumi-1 cells transduced with wt-FTO, mut-FTO, or control vector (**H**); or with *FTO* shRNA or shNS vectors (**I**). **J** Dual luciferase reporter assays for the effect of wild-type or mutant FTO on the relative luciferase activity of psiCHECK2-IGFBP2-3′-UTR with either wild-type or mutant m^6^A sites. **K**, **L** The mRNA half-life (t_1/2_) of *IGFBP2* transcripts in SKNO-1 and Kasumi-1 cells transduced with FTO shRNA or shNS vectors (**K**) or treated with DMSO or FB23-2 for 72 h (**L**). **M**–**O** RIP-qPCR showing the interaction between *IGFBP2* mRNA transcripts and YTHDF1 (**M**), YTHDF2 (**N**) and YTHDF3 (**O**) protein in SKNO-1 and Kasumi-1 cells. **P**, **Q** Increased expression of IGFBP2 after YTHDF1, YTHDF2 and YTHDF3 knockdown analyzed by qPCR (**P**) and western blotting (**Q**) in SKNO-1 and Kasumi-1 cells

Overexpression of wild-type FTO upregulated the expression of IGFBP2 at both the mRNA and protein levels compared with those in control and mutant FTO cells, whereas silencing the endogenous expression of FTO resulted, as expected, to an opposite pattern (Fig. 6A–D). In addition, treatment with FB23-2 also resulted in a dose-dependent reduction in the expression of IGFBP2 (Fig. 6E, F). RIP assay demonstrated the direct binding of FTO to *IGFBP2* transcripts (Fig. 6G). Gene-specific m^6^A qPCR assays further confirmed that forced expression of wild-type FTO substantially reduced the levels of m^6^A in *IGFBP2* mRNA transcripts (Fig. 6H); opposite results were shown when the endogenous FTO was knocked down (Fig. 6I). To further validate that FTO targets the 3′-UTR of *IGFBP2* mRNA transcripts, we created reporter vectors baring the coding sequence of *IGFBP2* 3′-UTR or corresponding m^6^A consensus mutant sequences (Additional file 1: Fig. S6A) and found that compared with the empty and mutant FTO vectors, luciferase activity was significantly promoted by wild-type FTO when cotransfected with the reporter vector containing the wild-type *IGFBP2* 3′-UTR fragment with intact m^6^A consensus, whereas this effect was abrogated when cotransfected with reporter vectors containing mutant m^6^A consensuses (Fig. 6J).

RNA stability assays indicated that *FTO* knockdown noticeably shortened the half-life of *IGFBP2* mRNA transcripts in SKNO-1 and Kasumi-1 cells (Fig. 6K). Likewise, the half-life of *IGFBP2* mRNA transcripts manifested a dose-dependent reduction when the two t(8;21) AML cells were treated with FB23-2 (Fig. 6L), suggesting that upregulation of IGFPB2 is at least partially attributed to the increased stability of its transcripts mediated by FTO-induced m^6^A demethylation.

To screen specific m^6^A readers targeting the 3′-UTR of *IGFBP2*, we performed an RNA pull-down assay using 4 pairs of m^6^A methylated or unmethylated single-stranded RNAs carrying part of the 3′-UTR sequence of *IGFBP2* (~ 20 bp in length) as baits, followed by mass spectrometry analysis*.* Each pair of RNA baits contained one of the 4 putative m^6^A consensus motifs in the 3′-UTR of *IGFBP2* (Additional file 1: Fig. S6B). We detected that the m^6^A reader YTHDF2 was dramatically enriched in all 4 m^6^A-modified RNA baits, YTHDF1 and YTHDF3 were markedly enriched in 3 of the 4 m^6^A-methylated baits compared with the unmethylated control, whereas other known readers were enriched in no more than 1 bait (YTHDC2, IGF2BP2/3, HNRNPA2B1 and HNRNPC were enriched in only 1 baits and YTHDC1/3, IGF2BP1 were enriched in none of the 4 baits; Additional file 1: Fig. S6C). RIP assays further confirmed that YTHDF1, YTHDF2 and YTHDF3 bound to *IGFBP2* transcripts directly (Fig. 6M–O). The expression of IGFBP2 was barely altered when either YTHDF2 or YTHDF3 was knocked down (Additional file 1: Fig. S6D and S6E). However, when the three YTHDF proteins were simultaneously knockdown by shRNA, the expression of IGFBP2 were greatly increased (Fig. 6P, Q), suggesting that the YTHDFs proteins work together to mediate the decay of IGFBP2 mRNA.

### IGFBP2 is a functionally important target gene of FTO in t(8;21) AML

**Fig. 7:**
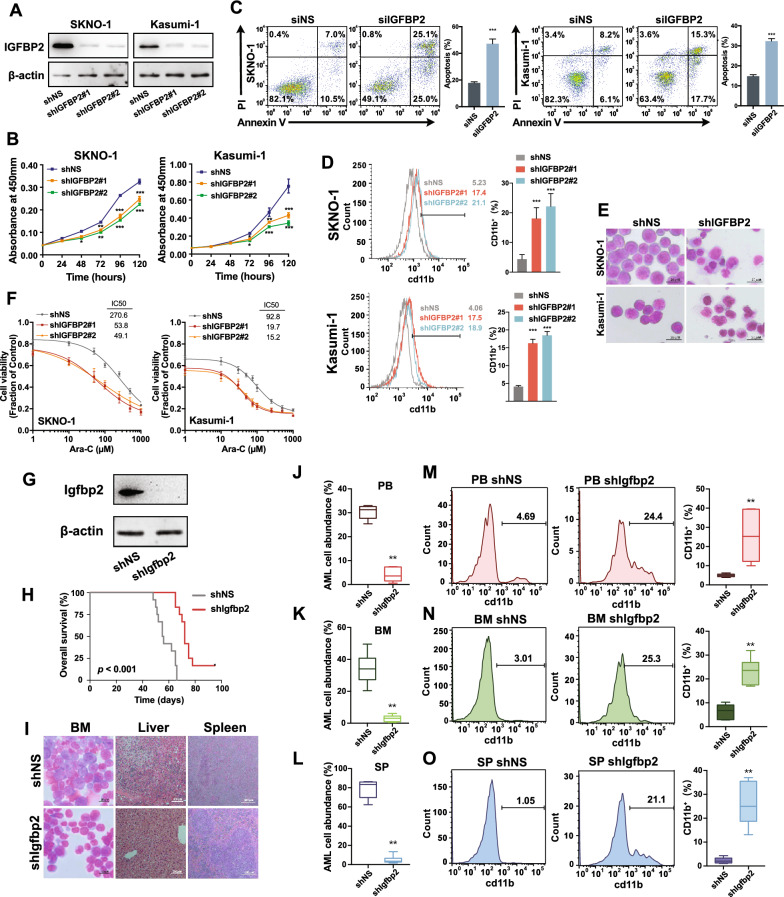
IGFBP2 promotes leukemogenesis and resistance to Ara-C. **A** Confirmation of IGFBP2 knockdown by shRNAs in SKNO-1 and Kasumi-1 cells using western blotting. **B** Effects of *IGFBP2* knockdown on the proliferation of SKNO-1 and Kasumi-1 cells by CCK8 assays. **C** Effect of silencing *IGFBP2* on cell apoptosis in SKNO-1 and Kasumi-1 cells 72 h after siRNA transfection. **D** The effect of IGFBP2 knockdown on differentiation of SKNO-1 and Kausmi-1 cells. The percentage of CD11b^+^ cells was quantified (right panel). **E** Wright-Giemsa staining of SKNO-1 and Kausmi-1 cells with or without IGFBP2 knockdown. **F** CCK-8 assays for SKNO-1 and Kasumi-1 cells transfected with IGFBP2 shRNA or scramble vectors treated with varying concentrations of Ara-C for 48 h. **G** Western blot analysis for knockdown of *Igfbp2* in AML1-ETO9a-driven AML cells. **H** Kaplan–Meier survival curves of AML1-ETO9a-driven AML mice (n = 10 for each group) after *Igfbp2* knockdown. **I** Wright-Giemsa staining of bone marrow and H&E staining of livers and spleens for Igfbp2-knockdown AML1-ETO9a-driven AML mice. **J**–**O** Flow cytometric analysis of the percentage of GFP^+^ AML1-ETO9a AML cells (**J**–**L**) and distribution of anti-CD11b-stained GFP + AML cells (**M**–**O**) in PB, BM, and SP of AML1-ETO9a-driven AML mice with and without *Igfbp2* knockdown 7 weeks after transplantation

The pathological role of IGFPB2 in t(8;21) AML was then investigated. When endogenous IGFBP2 was knocked down in the two t(8;21) AML cell lines (Fig. 7A), we observed a substantial repression on cell proliferation (Fig. 7B) and colony-forming capacity (Additional file 1: Fig. S7A) and a promotion on G1 cell cycle arrest (Additional file 1: Fig. S7B), cell apoptosis (Fig. 7C and Additional file 1: Fig. S7C) and cell differentiation (Fig. 7D, E), which was largely similar to the effect of the *FTO* knockdown. Moreover, IGFBP2 knockdown notably decreased the IC_50_ value of Ara-C in the two Ara-C resistant cell lines (Fig. 7F).

In the AML1-ETO9a AML mouse model, we found that *Igfbp2* knockdown prolonged the overall survival of recipient mice (log-rank test, *p* < 0.001; Fig. 7G, H). Leukemic infiltration in BM, liver and spleen was also significantly suppressed when *Igfbp2* was knocked down (Fig. 7I and Additional file 1: Fig. S7D–E). Moreover, *Igfbp2* knockdown decreased the abundance of leukemic cells (Fig. 7J–L) and the proportion of CD11b-stained leukemic cells (Fig. 7M–O) in PB, BM, and spleen samples.

**Fig. 8:**
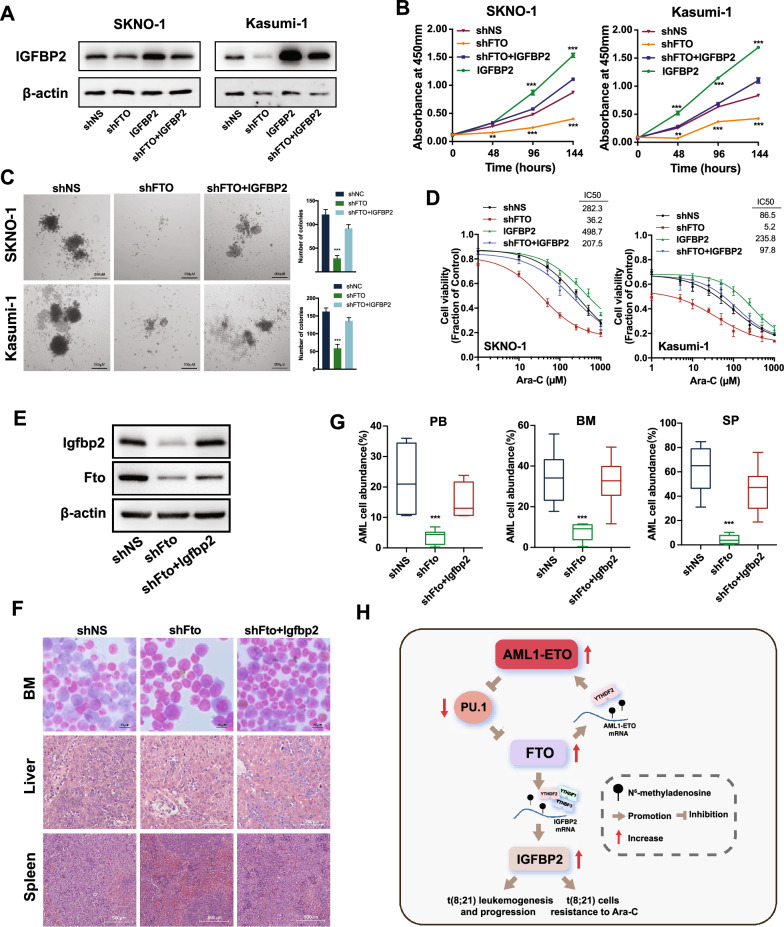
FTO regulates leukemogenesis and sensitivity of t(8;21) AML cells to Ara-C through IGFBP2. **A** Western blotting for the expression of IGFBP2 in SKNO-1 and Kasumi-1 cells transduced with shNS (pLKO.1-shNS + empty pTSB), shFTO (pLKO.1-shFTO#1 + empty pTSB), shFTO + IGFBP2 (pLKO.1-shFTO#1 + pTSB-IGFBP2-CDS), or IGFBP2 (pLKO.1-shNS + pTSB-IGFBP2-CDS). shNS, scramble shRNA. **B** CCK-8 assays for the effects of *FTO* knockdown and/or overexpression of *IGFBP2* on the proliferation of SKNO-1 and Kasumi-1 cells. **C** Effects of *FTO* knockdown with *IGFBP2* overexpression on colony-forming capacity of SKNO-1 and Kasumi-1 cells. **D** CCK-8 assays for the effects of *FTO* knockdown and/or overexpression of *IGFBP2* on the sensitivity to Ara-C in SKNO-1 and Kasumi-1 cells. **E**–**G** Effects of *FTO* knockdown with *IGFBP2* overexpression on AML1-ETO9a-driven AML mice. Western blot analysis for *Igfbp2 and Fto* (**E**), Wright-Giemsa staining of bone marrow and H&E staining of livers and spleens (**F**) and flow cytometric analysis of the abundance of GFP^+^ AML1-ETO9a AML cells in PB, BM and SP (**G**) were shown. **H** Proposed model depicting the regulatory interactions and role of FTO in leukemogenesis and Ara-C resistance in t(8;21) AML.

Rescue experiments demonstrated that when IGFBP2 was forced to be overexpressed in FTO-knockdown t(8;21) AML cells (Fig. 8A), the restoration of cell proliferation (Fig. 8B), colony-forming capacity (Fig. 8C) and regain of Ara-C tolerance (Fig. 8D and Additional file 1: Fig. S8A) were observed. Rescue experiments conducted with AML1-ETO9a AML mouse model also revealed a significant restoration in the infiltration of leukemic cells in PB, BM, liver and spleen when Igfbp2 was overexpressed in Fto-knockdown cells (Fig. 8E–G and Additional file 1: Fig. S8B–C). Force expression of Igfbp2 greatly prevented differentiation of leukemic cells in PB, BM and spleen mediated by Fto knockdown (Additional file 1: Fig. S8D). Taken together, our data suggest that IGFBP2 is a functionally critical target of FTO and is essential for FTO-mediated t(8;21) AML progression and Ara-C resistance.

## Discussion

In the present study, we demonstrate a positive regulatory loop between the fusion protein AML1-ETO and FTO, which exerts a critical role in promoting leukemogenesis and resistance of t(8;21) AML cells to Ara-C by modulating the expression of its mRNA targets, such as IGFBP2, via m^6^A demethylation (see the proposed model in Fig. 8H).

Although the overexpression of FTO are found in several AML subtypes, and it has been reported that MLL fusions may upregulate the expression of FTO by directly targeting it in MLL-rearranged AML cells [18], the underlying molecular mechanism driving FTO overexpression in t(8;21) AML remains to be determined. AML1-ETO has been shown to promote or silence the expression of various oncogenes or cancer suppressor genes via genetic or epigenetic regulation [6, 7]. In the present study, although no evidence was found to indicate a direct interaction between the AML1-ETO protein and *FTO* gene, the transcription factor PU.1, which is critical during myelopoiesis, might play a bridging role between AML1-ETO and FTO. PU.1 has been reported to strongly promote differentiation of myeloid lineages [43, 44], acting as a tumor suppressor gene in AML [45]. Moreover, PU.1 is known to be expressed at a low level in t(8;21) AML [34, 46]. AML1-ETO has been reported to inhibit the transcriptional capacity of PU.1 by expelling the coactivator c-Jun from the β_3_β_4_ region of PU.1 [34]. Our findings indicate that AML1-ETO can also repress the expression of PU.1 via binding to its promoter, thereby inhibiting the PU.1-mediated transcriptional repression of FTO. The AML1-ETO/PU.1 axis may be responsible for the high expression of FTO in t(8;21) AML.

The pivotal role of AML1-ETO in the pathogenesis of t(8;21) AML makes it a promising candidate for therapeutic intervention [47]. Moreover, the copy number of AML1-ETO transcripts has been regarded as an indicator of relapse in t(8;21) AML [48]. Given the challenge of directly targeting AML1-ETO, it would be worthwhile to investigate the molecular mechanisms that govern its expression. Our findings show that AML1-ETO is also a downstream target of FTO; this regulation is achieved at least partially due to the inhibition of *AML1-ETO* mRNA decay mediated by YTHDF2. Of note, a study reported that AML1-ETO can repress the transcription of YTHDF2 via targeting its promoter [23], while our results reveal that YTHDF2 inhibits the expression of AML1-ETO in a m^6^A-dependent manner. Thus, these results suggest a negative feedback loop between AML1-ETO and YTHDF2 that maintains the stable expression level of the two proteins.

Although the oncogenic role of FTO has been reported in leukemia, the functional significance of FTO in some leukemic cells, such as the human erythroleukemic cell line K562, appears to be less essential, possibly due to a lower level of endogenous expression in these cells [18]. The higher level of FTO which may be attributed to the AML1-ETO/FTO feedback loop and the in vitro and in vivo evidence further validate the essential role of FTO in promoting leukemogenesis and progression of t(8;21) AML. Furthermore, the involvement of FTO in Ara-C resistance is also investigated in the present study. Besides the high level of FTO mRNA in patients with primary refractory t(8;21) AML, our data demonstrate an intriguing finding that indicates sustained high levels of FTO mRNA in patients with *c-KIT* mutation even after complete remission. It is well appreciated that t(8;21) patients with *c-KIT* mutations had a significantly higher rate of relapse [29]. In addition, the acquired drug resistance and relapse is largely associated with the genetic heterogeneity within cancer cell clones. It is plausible to speculate that the post-chemotherapy surviving *c-KIT* mutations harboring clones, which are responsible for later relapse, express high levels of FTO. All these findings provide evidence of a strong relevance between FTO and chemoresistance in t(8;21) AML. Our present findings validate a substantial Ara-C resistance in the t(8;21) AML cell lines SKNO-1 and Kasumi-1, as evidenced by the IC_50_ values of approximately 1000-fold higher compared to the Ara-C sensitive cell lines HL-60 and HEL, which exhibited IC_50_ values of around 10^–1^ μM [49]. Suppression of FTO genetically or pharmacologically significantly sensitizes the two Ara-C-resistant cell lines to Ara-C, suggesting a potentially druggable target of FTO in the treatment of Ara-C resistance in t(8;21) AML. In addition to FB23-2, the newly developed small-molecule FTO inhibitors CS1 and CS2 have been reported to display effective antitumor effects even at low nanomolar levels [50]. Selective inhibition of FTO can break the AML1-ETO/FTO feedback loop, inhibit progression and resensitizes resistant cells to Ara-C, highlighting a great therapeutic potential for the efficacious small-molecule FTO inhibitors, in combination with routine chemotherapy, in the treatment of t(8;21) AML.

Accumulating evidence indicates that high expression of IGFBP2 promotes tumorigenesis, progression, and chemotherapy resistance in many malignant tumors [51–53]. Studies in AML also demonstrate that IGFBP2 supports the activity of hematopoietic stem cells (HSCs) and promotes the proliferation of AML cells by suppressing PTEN expression and activating Akt [42, 54]. High expression of IGFBP2 is also found to be an independent predictive factor for primary refractory AML, suggesting a potential relationship between IGFBP2 and chemoresistance in AML [54]. Concordantly, our study reveals that IGFBP2, as an important target of FTO, exerts a crucial role in both cell proliferation and Ara-C resistance in t(8;21) AML. In addition, the Akt activation has been reported to be implicated in drug resistance in multiple cancers [55]. It might serve as a potential mechanism underlying IGFBP2-mediated Ara-C resistance, which requires further confirmation.

It is worth noting that nearly two-thirds of mRNA transcripts with hypomethylated m^6^A peaks after overexpression of FTO demonstrate upregulated mRNA levels in t(8;21) AML cells, contradicting the results in the MONOMAC-6 AML cell line in a previous study [18]. This indicates the heterogeneity of FTO-dependent m^6^A modification patterns in different AML subtypes. It also suggests that FTO-mediated m^6^A demethylation in t(8;21) AML cells tends to enhance the mRNA stability of the majority FTO target genes, the process of which is likely to be mediated by YTHDF2/YTHDF3 [35, 56]. Our data demonstrate that the half-life of *IGFBP2* mRNA is shortened when *FTO* is knocked down. However, neither silencing YTHDF2 nor YTHDF3 significantly alters the expression of IGFBP2, although we show the direct binding of these 2 readers to the 3′-UTR of *IGFBP2* mRNA. Interestingly, a recent study reports that YTHDFs family proteins (including YTHDF1, YTHDF2, and YTHDF3) tend to bind the same m^6^A-methylated mRNAs and function together to promote mRNA degradation. The ability to regulate mRNA decay becomes evident when all 3 YTHDFs proteins are simultaneously depleted [57]. Similarly, our data indicate that the three YTHDFs, rather than other known m^6^A readers, can be enriched by the m^6^A methylated single-stranded RNA of the *IGFBP2* 3′-UTR. The expression of IGFBP2 is upregulated when the three YTHDFs are knockdown simultaneously, indicating that YTHDFs proteins work together to mediate the decay of *IGFBP2* mRNA in t(8;21) AML.

## Conclusion

In summary, we reveal a positive feedback loop between the leukemia-initiating fusion protein AML1-ETO and the m^6^A demethylase FTO in t(8;21) AML. AML1-ETO upregulates the expression of FTO through PU.1; FTO in turn promotes the expression of AML1-ETO by inhibiting YTHDF2-mediated *AML1-ETO* mRNA decay through m^6^A demethylation. The use of FTO selective inhibitors effectively represses t(8;21) AML progression, promotes cell differentiation and sensitizes resistant cells to Ara-C. The AML1-ETO/FTO/IGFBP2 minicircuitry holds potential as a therapeutic target in t(8;21) AML, particularly for the Ara-C tolerant patients.

### Supplementary Information

**Additional file 1: Fig. S1.** AML1-ETO promotes expression of FTO via PU.1. (A) Comparison of overall survival of patients with de novo t(8;21) AML (n = 26) using the Kaplan–Meier method grouped by the expression of FTO (high vs. low). *p *value was evaluated using the log-rank test. (B) Kaplan–Meier analysis of event-free survival (left) and overall survival (right) of patients with AML (n = 344, data from GSE6891) based on the expression of *FTO*. (C) Comparison of the expression of *FTO* in Kasumi-1 cells with or without AML1-ETO knockdown (shAE vs. shNS) detected by RNA-seq in the GSE115121 data set. (D) ChIP-seq of GSE65427 depicting *FTO* loci in Kasumi-1 cells targeting C- terminus of ETO (upper panel) and N-terminus of AML1(lower panel), representing AML1-ETO peaks on *FTO*. (E) Schematic diagrams showing the amplified regions on the promoter of *SPI1* for the ChIP-qPCR showed in Fig. 1I and Fig. S1F. The location of targeted amplified region (named ‘Target’) and negative control site (NC) are indicated with blue horizontal lines. The red triangle indicates the location of peak summit of AML1-ETO on the promoter of *SPI1* detected by the ChIP-seq of GSE65427. (F) ChIP-qPCR assays showing no direct binding of AML1 or ETO within 200 bp upstream of the *SPI1* promoter in SKNO-1-siAE cells. (G and H) Pearson correlation of the expression of *FTO* and *SPI1* in (G) normal blood tissues from the Genotype-Tissue Expression Project (GTEx, n = 444) or (H) BM samples of patients with AML from TCGA database (n = 173). (I) Sequences of the 4 *FTO* promoter fragments (the P1 to P4 showed in Fig. 1P) and putative PU.1 binging sites (sites 1, 2, and 3). **Fig. S2.** FTO upregulated AML1-ETO in a m^6^A-dependent manner. (A–C) The level of *AML1-ETO* mRNA detected by qPCR in SKNO-1 and Kasumi-1 cells (A) transduced with wild-type FTO (wt-FTO), mutant FTO (mut-FTO), or mock vectors; (B) transduced with *FTO*-knockdown (shFTO#1 and shFTO#2) or scramble shRNA (shNS) vectors; (C) treated with DMSO or FB23-2 treatment for 72 h. **Fig. S3.** Oncogenic role of FTO in t(8;21) AML cells and AML1-ETO9a driven AML mice. (A) Effects of forced expression or knockdown of FTO on cell cycle in SKNO-1 and Kasumi-1 cells. (B) Effects of forced expression or knockdown of FTO on colony-forming capacity of Kasumi-1 cells. (C) The effect of FTO knockdown on differentiation of Kasumi-1 cells. The percentage of CD11b^+^ cells was quantified (right panel). (D) Wright-Giemsa staining of Kasumi-1 cells with or without FTO knockdown. (E) Spleen size in AML1-ETO9a-driven AML mice with or without *Fto* knockdown or treatment with DMSO or FB23-2 (6 mg/kg) 7 weeks after transplantation (n = 6 for each group). (F) Spleen weight of AML1-ETO9a-driven AML mice from (E). (G) Wright-Giemsa staining of bone marrow of AML1-ETO9a-driven AML mice. (H–J) Percentage of GFP^+^ AML1-ETO9a AML cells in the (H) peripheral blood (PB), (I) bone marrow (BM), and (J) spleen (SP) of the mice treatment with DMSO or FB23-2 by flow cytometric analysis. (K–M) Flow cytometric analysis of the distribution of anti-CD11b-stained GFP + AML1-ETO9a AML cells in PB (K), BM (L), and SP (M) of mice treatment with DMSO or FB23-2. **Fig. S4.** Suppression of FTO resensitizes resistant cells to Ara-C in vitro and in vivo. (A and B) Apoptosis measured by flow cytometry for SKNO-1 (A) and Kasumi-1 (B) cells treated with DMSO, 30 µM Ara-C alone, 10 µM FB23-2 alone or combination of Ara-C and FB23-2 for 48 h with FB23-2 pretreatment for 6 h. (C and D) Percentage of GFP^+^ AML cells in peripheral blood (C) and spleen (D) of NOD/SCID/γ_c_^null^ immunodeficient mice injected with SKNO-1 cells through tail vein treated with DMSO, Ara-C, FB23-2, or a combination of Ara-C and FB23-2 (n = 6 for each group). **Fig. S5.** Transcriptome-wide identification of FTO targets in t(8;21) AML. (A and B) Proportion of the distribution of m^6^A peaks in exon, intron, and intergenic regions across entire mRNA transcripts (A) or in the 5′-UTR, first exon, other exon, and 3′-UTR of mRNA transcripts (B) detected by m^6^A-seq assays in Kasumi-1 cells transduced with wild-type FTO or empty vector. (C and D) Gene set enrichment analysis (GSEA) of genes with a significant decrease in m^6^A levels as well as a significant increase (Hypo-up) or decrease (Hypo-down) in overall transcript levels in FTO-overexpressing Kasumi-1 cells. (E) Comparison of *IGFBP2* expression between human primary AML cases with t(8;21) (n = 30, data from GSE30285) or normal controls (NC) (n = 9, data from GSE34814). (F) Kaplan–Meier analysis of event-free survival (left) and overall survival (right) of patients with AML (n = 344, data from GSE6891) based on the expression of *IGFBP2*. (G) Pearson correlation of the expression of *FTO* and *IGFBP2* in BM samples of patients with AML from TCGA database (n = 173). **Fig. S6.** Luciferase reporter construction and identification of specific m^6^A readers targeting the 3′-UTR of *IGFBP2* mRNA. (A) Construction of luciferase reporter vectors. Synthesized wildtype (wt) or mutant (mut) 3′ coding sequences of *IGFBP2* were inserted into the XhoI and NotI site of the psiCHECK2 luciferase reporter. Putative m^6^A consensus motifs are shown in bold, whereas mutation sites (A to T mutation) are shown in red. (B and C) Identification of m^6^A specific binding proteins on 3′-UTR of *IGFBP2* by RNA pull-down using 4 pairs of single-stranded RNA (ssRNA) baits containing the 4 m^6^A consensus motif on the 3′-UTR sequence of *IGFBP2* respectively, with methylated (green) or unmethylated (red) adenosine (B). The iBAQ value of previously reported m^6^A readers (including YTHDFs, YTHDCs, IG2FBPs and hnRNPs) enriched by the 4 pairs of ssRNA probes detected by mass spectrometry analysis are shown (C). The YTHDC1, YTHDC3, and IGF2BP1 proteins that could not be enriched by all 4 pairs of ssRNA probes are not shown in the Figure (see Additional file 3: Table S4). (D and E) Western blot analysis of the expression of IGFBP2 with or without silencing of YTHDF2 (D) or YTHDF3 (E) by siRNA in SKNO-1 or Kasumi-1 cells. siNS, scramble siRNA. **Fig. S7.** Functional role of IGFBP2 in t(8;21) AML. (A and B) Effects of *IGFBP2* knockdown on colony-forming capacity (A) and cell cycle (B) in SKNO-1 and Kasumi-1 cells. (C) Western blot analysis of silencing *IGFBP2* by siRNA in SKNO-1 and Kasumi-1 cells. siNS, scramble siRNA. (D and E) External views (D) and weight (E) of the spleens from AML1-ETO9a-driven AML mice with or without *Igfbp2* knockdown (n = 6 for each group) 7 weeks after transplantation. **Fig. S8.** FTO regulates leukemogenesis and sensitivity of t(8;21) AML cells to Ara-C through IGFBP2. (A) Effects of *FTO* knockdown with *IGFBP2* overexpression after Ara-C treatment on colony-forming capacity of SKNO-1 and Kasumi-1 cells. (B–D) External views (B), weight of the spleens (C) and flow cytometric analysis of CD11b^+^ AML cells in PB, BM, and SP (D) of AML1-ETO9a-driven AML mice in Fig. 8E–G.

**Additional file 2: Table S1.** The clinical characteristics, morphological and genetic features of patients with de novo t(8;21) AML at diagnosis (n = 26) and relapse (n = 1). **Table S2.** List of 18 genes that show significant decrease abundance in m^6^A peak and significant change in the levels of corresponding mRNA transcript between FTO-overexpressing and control Kasumi-1 cells, and are also significantly positively or negatively correlated with FTO in expression in TCGA AML database. **Table S3.** (Related to Methods) List of oligonucleotides.

**Additional file 3: Table S4**. (Related to Fig. S6C**)** Results of mass spectrometry analysis for the proteins obtained in RNA pull down assay.

## Data Availability

The original contributions presented in the study are included in the article/supplementary material, further inquiries can be directed to the corresponding authors.

## Abbreviations

AML: Acute myeloid leukemia
m^6^A: N^6^-methyladenosine
FTO: Fat mass and obesity-associated protein
YTHDF2: YTH domain family 2
IGFBP2: Insulin-like growth factor binding protein 2
BM: Bone marrow
PB: Peripheral blood
TSS: Transcription start site
shNS: Scramble shRNA
3′-UTR: 3′ Untranslated region
IgG: Immunoglobulin G
RIP: RNA immunoprecipitation
m^6^A-seq: m^6^A sequencing
RNA-seq: RNA-sequencing
ChIP-seq: Chromatin immunoprecipitation sequencing
CR: Complete remission
TCGA: The Cancer Genome Atlas
