# Supplementary material for: A novel AML1-ETO/FTO positive feedback loop promotes leukemogenesis and Ara-C resistance via stabilizing IGFBP2 in t(8;21) acute myeloid leukemia
Source: Exp Hematol Oncol. 2024 Jan 24;13:9. doi: 10.1186/s40164-024-00480-z (PMC10807068; doi:10.1186/s40164-024-00480-z)
Supplement: Supplementary file 1 — Additional file 1: Fig. S1. AML1-ETO promotes expression of FTO via PU.1. (A) Comparison of overall survival of patients with de novo t(8;21) AML (n = 26) using the Kaplan–Meier method grouped by the expression of FTO (high vs. low). p value was evaluated using the log-rank test. (B) Kaplan–Meier analysis of event-free survival (left) and overall survival (right) of patients with AML (n = 344, data from GSE6891) based on the expression of FTO. (C) Comparison of the expression of FTO in Kasumi-1 cells with or without AML1-ETO knockdown (shAE vs. shNS) detected by RNA-seq in the GSE115121 data set. (D) ChIP-seq of GSE65427 depicting FTO loci in Kasumi-1 cells targeting C- terminus of ETO (upper panel) and N-terminus of AML1(lower panel), representing AML1-ETO peaks on FTO. (E) Schematic diagrams showing the amplified regions on the promoter of SPI1 for the ChIP-qPCR showed in Fig. 1I and Fig. S1F. The location of targeted amplified region (named ‘Target’) and negative control site (NC) are indicated with blue horizontal lines. The red triangle indicates the location of peak summit of AML1-ETO on the promoter of SPI1 detected by the ChIP-seq of GSE65427. (F) ChIP-qPCR assays showing no direct binding of AML1 or ETO within 200 bp upstream of the SPI1 promoter in SKNO-1-siAE cells. (G and H) Pearson correlation of the expression of FTO and SPI1 in (G) normal blood tissues from the Genotype-Tissue Expression Project (GTEx, n = 444) or (H) BM samples of patients with AML from TCGA database (n = 173). (I) Sequences of the 4 FTO promoter fragments (the P1 to P4 showed in Fig. 1P) and putative PU.1 binging sites (sites 1, 2, and 3). Fig. S2. FTO upregulated AML1-ETO in a m6A-dependent manner. (A–C) The level of AML1-ETO mRNA detected by qPCR in SKNO-1 and Kasumi-1 cells (A) transduced with wild-type FTO (wt-FTO), mutant FTO (mut-FTO), or mock vectors; (B) transduced with FTO-knockdown (shFTO#1 and shFTO#2) or scramble shRNA (shNS) vectors; (C) treated with DMSO or FB23-2 trea [file 40164_2024_480_MOESM1_ESM.pdf]

SUPPLEMENTARY FIGURES

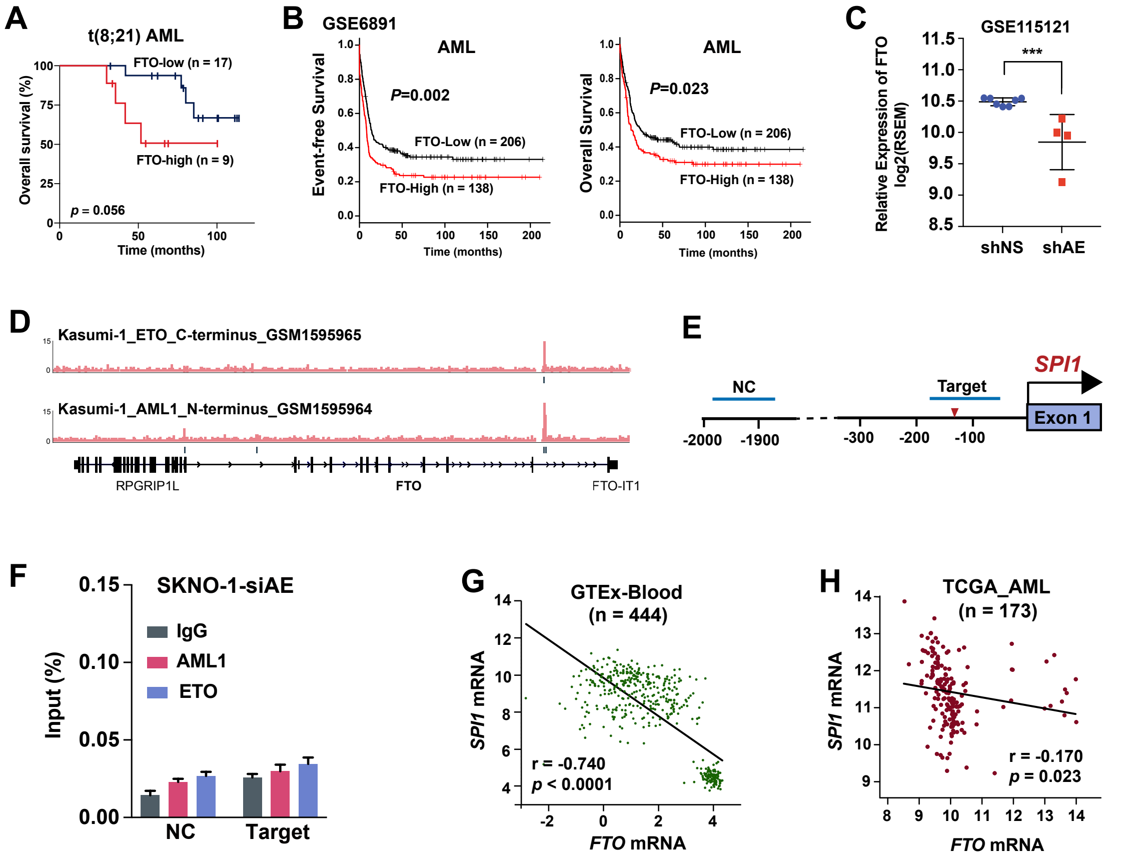

I

P1

GGAGAAACATGGCAGGCTCCCGTAGCCTCCT**GGGAAA**TGTAGTTCTCCTTGGACTCTAGCCTGTTTGC
TCGCGGGGTAGCGGACTACGCTCTTCCAGCTGTCGGACCT**GGGAAA**TTCTCCTGTGCTAAATCCCGTG
GCGCTCGCGGGTGTCGCCGCGGTGCATCCTGGGAGTTGTAGTTTTTTCTACTCAGAGGGAGAATAGCT
CCAGACGGGAGCAGGACGCTGA**GAGAAC**TACATGCAGGAGGCGGGGTCCAGGGCGAGGGATCTACG
CAGCTTGCGGTGGCGAAGGCGGCTTTAGTGGCAGC

P2

GTTTGCTCGCGGGGTAGCGGACTACGCTCTTCCAGCTGTCGGACCT**GGGAAA**TTCTCCTGTGCTAAAT
CCCGTGGCGCTCGCGGGTGTCGCCGCGGTGCATCCTGGGAGTTGTAGTTTTTTCTACTCAGAGGGAG
AATAGCTCCAGACGGGAGCAGGACGCTGA**GAGAAC**TACATGCAGGAGGCGGGGTCCAGGGCGAGGG
ATCTACGCAGCTTGCGGTGGCGAAGGCGGCTTTAGTGGCAGC

P3

GTTTTTTCTACTCAGAGGGAGAATAGCTCCAGACGGGAGCAGGACGCTGA**GAGAAC**TACATGCAGGAG
GCGGGGTCCAGGGCGAGGGATCTACGCAGCTTGCGGTGGCGAAGGCGGCTTTAGTGGCAGC

P4

GCGGGGTCCAGGGCGAGGGATCTACGCAGCTTGCGGTGGCGAAGGCGGCTTTAGTGGCAGC

Fig. S1. (related to Fig. 1) AML1-ETO promotes expression of FTO via PU.1. (A) Comparison of overall survival of patients with de novo t(8;21) AML (n = 26) using the Kaplan-Meier method grouped by the expression of FTO (high vs. low). *p*-value was evaluated using the log-rank test. (B) Kaplan-Meier analysis of event-free survival (left) and overall survival (right) of patients with AML (n = 344, data from GSE6891) based on the expression of *FTO*. (C) Comparison of the expression of *FTO* in Kasumi-1 cells with or without AML1-ETO knockdown (shAE vs. shNS) detected by RNA-seq in the GSE115121 data set. (D) ChIP-seq of GSE65427 depicting *FTO* loci in Kasumi-1 cells targeting C- terminus of ETO (upper panel) and N-terminus of AML1(lower panel), representing AML1-ETO peaks on *FTO*. (E) Schematic diagrams showing the amplified regions on the promoter of *SPI1* for the ChIP-qPCR showed in Fig. 1I and Fig. S1F. The location of targeted amplified region (named 'Target') and negative control site (NC) are indicated with blue horizontal lines. The red triangle indicates the location of peak summit of AML1-ETO on the promoter of *SPI1* detected by the ChIP-seq of GSE65427. (F) ChIP-qPCR assays showing no direct binding of AML1 or ETO within 200bp upstream of the *SPI1* promoter in SKNO-1-siAE cells. (G and H) Pearson correlation of the expression of *FTO* and *SPI1* in (G) normal blood tissues from the Genotype-Tissue Expression Project (GTEx, n = 444) or (H) BM samples of patients with AML from TCGA database (n = 173). (I) Sequences of the 4 *FTO* promoter fragments (the P1 to P4 showed in Fig. 1P) and putative PU.1 binging sites (sites 1, 2, and 3).

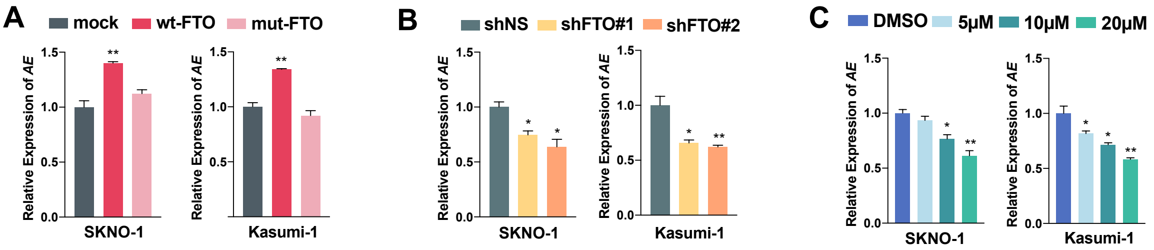

Fig. S2. (related to Fig. 2) FTO upregulated AML1-ETO in a m^{6}A-dependent manner. (A–C) The level of *AML1-ETO* mRNA detected by qPCR in SKNO-1 and Kasumi-1 cells (A) transduced with wild-type FTO (wt-FTO), mutant FTO (mut-FTO), or mock vectors; (B) transduced with *FTO*-knockdown (shFTO#1 and shFTO#2) or scramble shRNA (shNS) vectors; (C) treated with DMSO or FB23-2 treatment for 72 h.

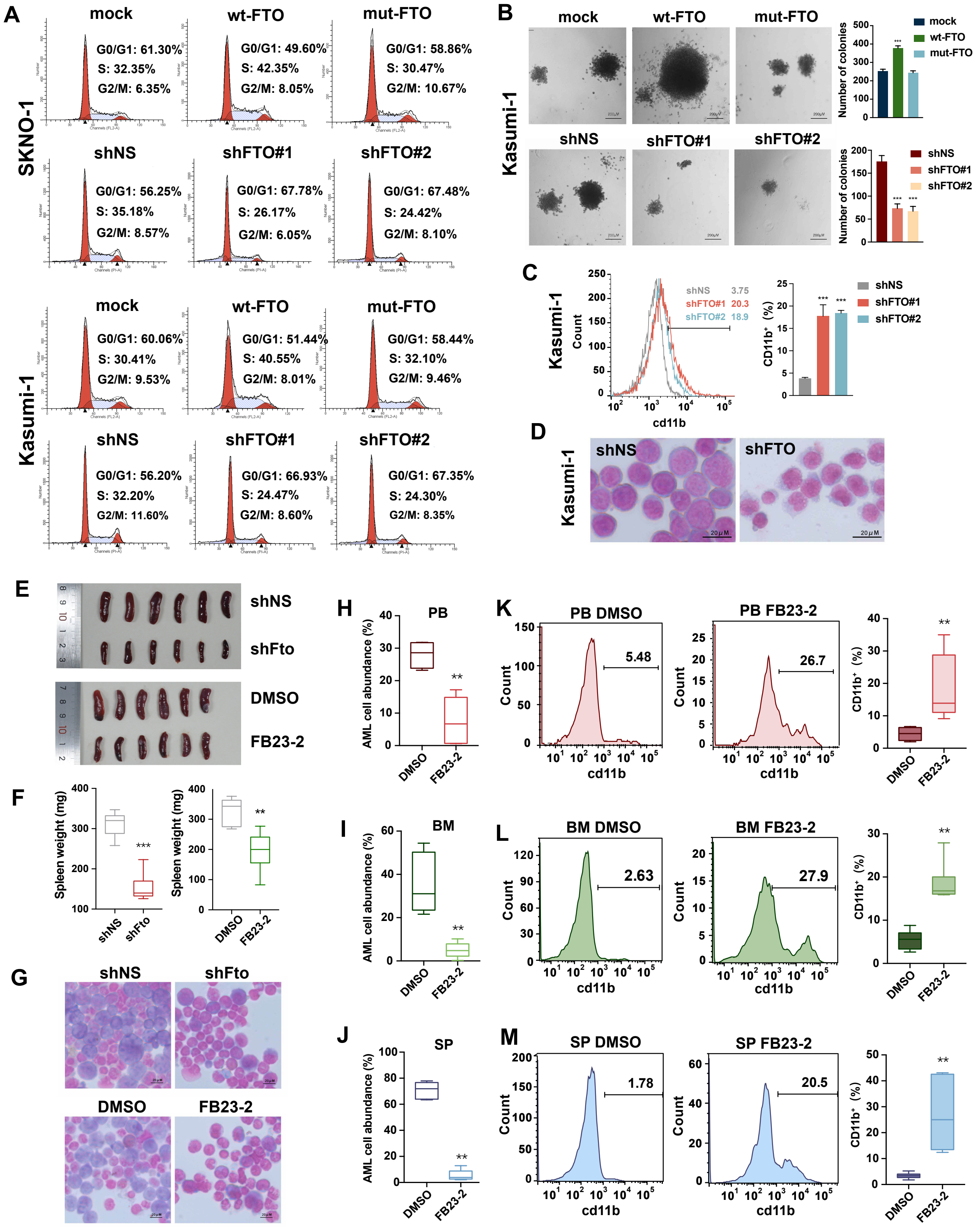
A
SKNO-1
mock
G0/G1: 61.30%
S: 32.35%
G2/M: 6.35%
wt-FTO
G0/G1: 49.60%
S: 42.35%
G2/M: 8.05%
mut-FTO
G0/G1: 58.86%
S: 30.47%
G2/M: 10.67%
shNS
G0/G1: 56.25%
S: 35.18%
G2/M: 8.57%
shFTO#1
G0/G1: 67.78%
S: 26.17%
G2/M: 6.05%
shFTO#2
G0/G1: 67.48%
S: 24.42%
G2/M: 8.10%
Kasumi-1
mock
G0/G1: 60.06%
S: 30.41%
G2/M: 9.53%
wt-FTO
G0/G1: 51.44%
S: 40.55%
G2/M: 8.01%
mut-FTO
G0/G1: 58.44%
S: 32.10%
G2/M: 9.46%
shNS
G0/G1: 56.20%
S: 32.20%
G2/M: 11.60%
shFTO#1
G0/G1: 66.93%
S: 24.47%
G2/M: 8.60%
shFTO#2
G0/G1: 67.35%
S: 24.30%
G2/M: 8.35%
B
Kasumi-1
mock
wt-FTO
mut-FTO
shNS
shFTO#1
shFTO#2
Number of colonies
mock
wt-FTO
mut-FTO
shNS
shFTO#1
shFTO#2
C
Kasumi-1
Count
cd11b
shNS 3.75
shFTO#1 20.3
shFTO#2 18.9
CD11b⁺ (%)
shNS
shFTO#1
shFTO#2
D
Kasumi-1
shNS
shFTO
20 μM
E
shNS
shFto
DMSO
FB23-2
F
Spleen weight (mg)
shNS
shFto
DMSO
FB23-2
G
shNS
shFto
DMSO
FB23-2
H
PB
AML cell abundance (%)
DMSO
FB23-2
I
BM
AML cell abundance (%)
DMSO
FB23-2
J
SP
AML cell abundance (%)
DMSO
FB23-2
K
PB DMSO
5.48
PB FB23-2
26.7
Count
cd11b
CD11b⁺ (%)
DMSO
FB23-2
L
BM DMSO
2.63
BM FB23-2
27.9
Count
cd11b
CD11b⁺ (%)
DMSO
FB23-2
M
SP DMSO
1.78
SP FB23-2
20.5
Count
cd11b
CD11b⁺ (%)
DMSO
FB23-2

Fig. S3. (related to Fig. 3) Oncogenic role of FTO in t(8;21) AML cells and AML1-ETO9a driven AML mice. (A) Effects of forced expression or knockdown of FTO on cell cycle in SKNO-1 and Kasumi-1 cells. (B) Effects of forced expression or knockdown of FTO on colony-forming capacity of Kasumi-1 cells. (C) The effect of FTO knockdown on differentiation of Kasumi-1 cells. The percentage of $CD11b^+$ cells was quantified (right panel). (D) Wright-Giemsa staining of Kasumi-1 cells with or without FTO knockdown. (E) Spleen size in AML1-ETO9a-driven AML mice with or without *Fto* knockdown or treatment with DMSO or FB23-2 (6 mg/kg) 7 weeks after transplantation (n = 6 for each group). (F) Spleen weight of AML1-ETO9a-driven AML mice from (E). (G) Wright-Giemsa staining of bone marrow of AML1-ETO9a-driven AML mice. (H–J) Percentage of GFP^+ AML1-ETO9a AML cells in the (H) peripheral blood (PB), (I) bone marrow (BM), and (J) spleen (SP) of the mice treatment with DMSO or FB23-2 by flow cytometric analysis. (K–M) Flow cytometric analysis of the distribution of anti-CD11b-stained GFP+ AML1-ETO9a AML cells in PB (K), BM (L), and SP (M) of mice treatment with DMSO or FB23-2.

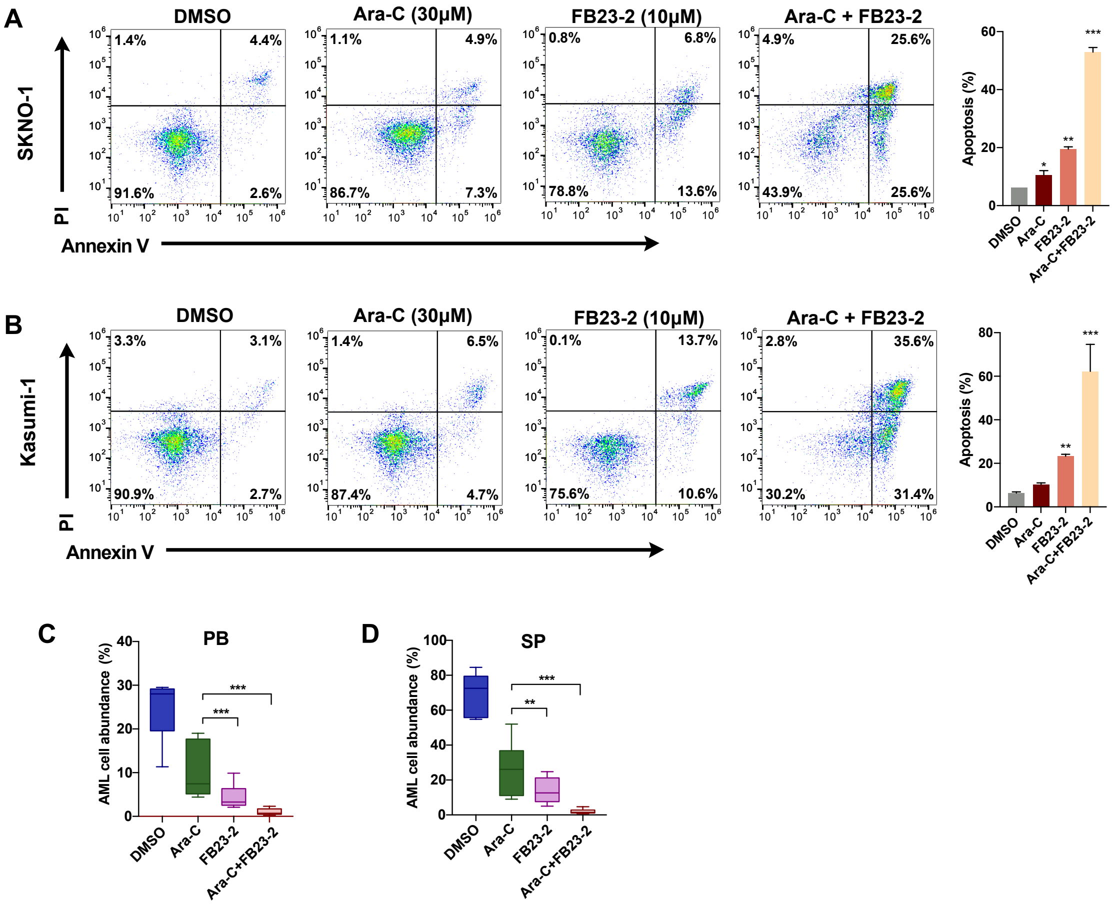

Fig. S4. (related to Fig. 4) Suppression of FTO resensitizes resistant cells to Ara-C in vitro and in vivo. (A and B) Apoptosis measured by flow cytometry for SKNO-1 (A) and Kasumi-1 (B) cells treated with DMSO, 30 µM Ara-C alone, 10 µM FB23-2 alone or combination of Ara-C and FB23-2 for 48 h with FB23-2 pretreatment for 6h. (C and D) Percentage of GFP^+ AML cells in peripheral blood (C) and spleen (D) of NOD/SCID/γ_c^{null} immunodeficient mice injected with SKNO-1 cells through tail vein treated with DMSO, Ara-C, FB23-2, or a combination of Ara-C and FB23-2 (n = 6 for each group).

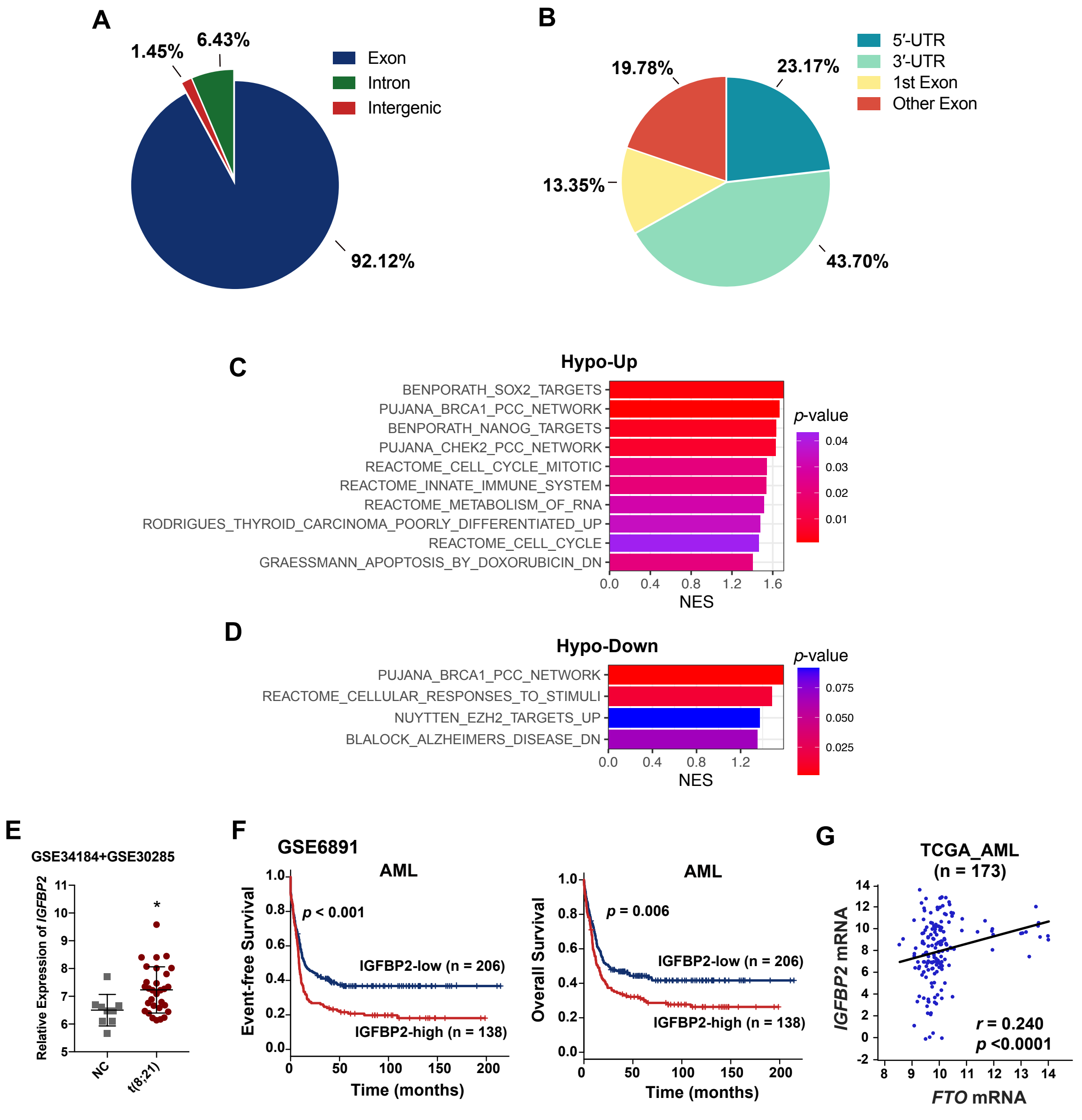

Fig. S5. (related to Fig. 5) Transcriptome-wide identification of FTO targets in t(8;21) AML. (A and B) Proportion of the distribution of m^{6}A peaks in exon, intron, and intergenic regions across entire mRNA transcripts (A) or in the 5′-UTR, first exon, other exon, and 3′-UTR of mRNA transcripts (B) detected by m^{6}A-seq assays in Kasumi-1 cells transduced with wild-type FTO or empty vector. (C and D) Gene set enrichment analysis (GSEA) of genes with a significant decrease in m^{6}A levels as well as a significant increase (Hypo-up) or decrease (Hypo-down) in overall

transcript levels in FTO-overexpressing Kasumi-1 cells. (E) Comparison of *IGFBP2* expression between human primary AML cases with t(8;21) (n = 30, data from GSE30285) or normal controls (NC) (n = 9, data from GSE34814). (F) Kaplan-Meier analysis of event-free survival (left) and overall survival (right) of patients with AML (n = 344, data from GSE6891) based on the expression of *IGFBP2*. (G) Pearson correlation of the expression of *FTO* and *IGFBP2* in BM samples of patients with AML from TCGA database (n = 173).

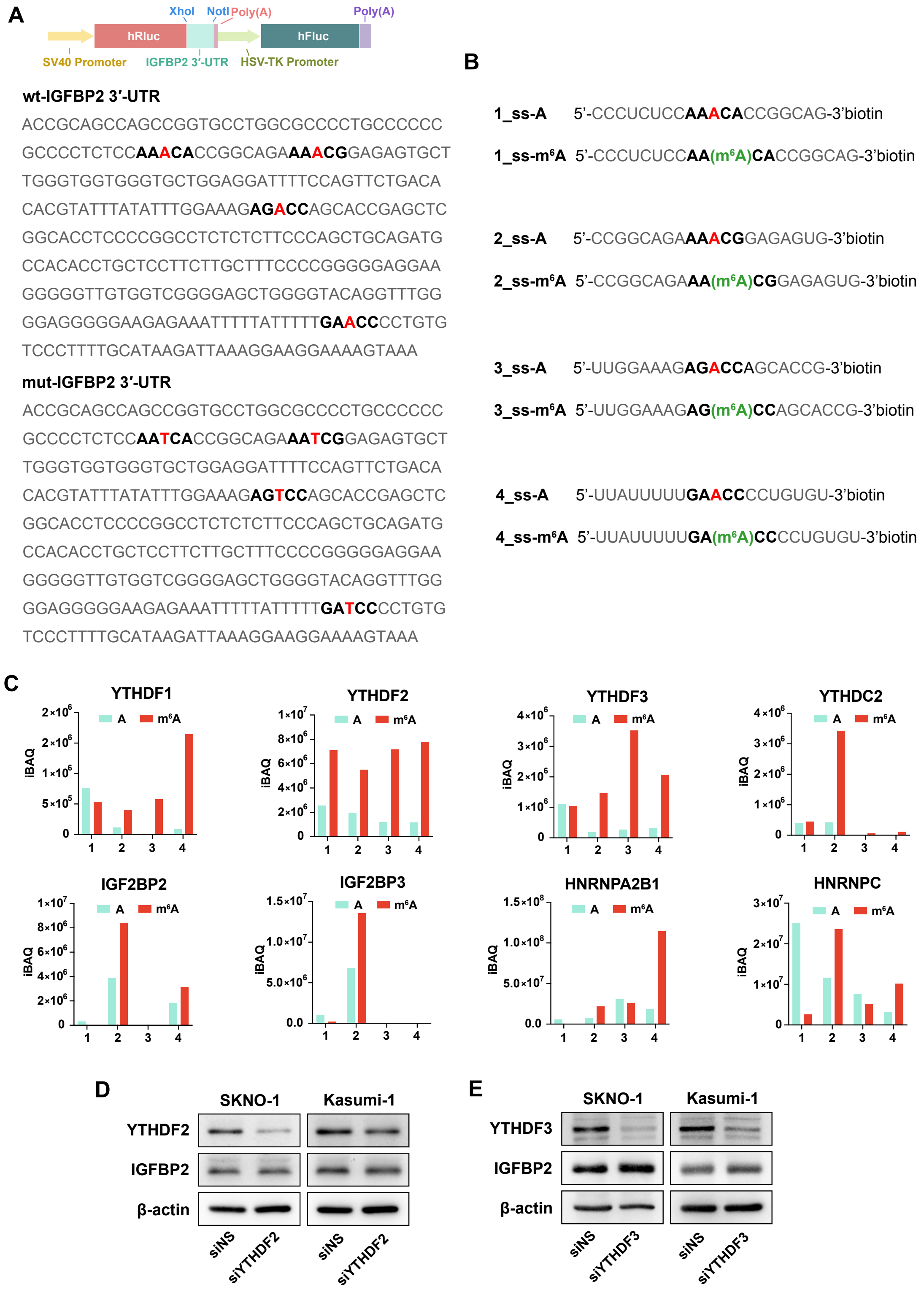
A
XhoI
NotI
Poly(A)
Poly(A)
hRluc
hFluc
SV40 Promoter
IGFBP2 3′-UTR
HSV-TK Promoter
wt-IGFBP2 3′-UTR
ACCGCAGCCAGCCGGTGCCTGGCGCCCCTGCCCCCC
GCCCCTCTCCAAACACCGGCAGAAAACGGAGAGTGCT
TGGGTGGTGGGTGCTGGAGGATTTTCCAGTTCTGACA
CACGTATTTATATTTGGAAAGAGACCAGCACCGAGCTC
GGCACCTCCCCGGCCTCTCTCTTCCCAGCTGCAGATG
CCACACCTGCTCCTTCTTGCTTTCCCCGGGGGAGGAA
GGGGGTTGTGGTCGGGGAGCTGGGGTACAGGTTTGG
GGAGGGGGAAGAGAAATTTTTATTTTTGAACCCCTGTG
TCCCTTTTGCATAAGATTAAAGGAAGGAAAAGTAAA
mut-IGFBP2 3′-UTR
ACCGCAGCCAGCCGGTGCCTGGCGCCCCTGCCCCCC
GCCCCTCTCCAATCACCGGCAGAAATCGGAGAGTGCT
TGGGTGGTGGGTGCTGGAGGATTTTCCAGTTCTGACA
CACGTATTTATATTTGGAAAGAGTCCAGCACCGAGCTC
GGCACCTCCCCGGCCTCTCTCTTCCCAGCTGCAGATG
CCACACCTGCTCCTTCTTGCTTTCCCCGGGGGAGGAA
GGGGGTTGTGGTCGGGGAGCTGGGGTACAGGTTTGG
GGAGGGGGAAGAGAAATTTTTATTTTTGATCCCCTGTG
TCCCTTTTGCATAAGATTAAAGGAAGGAAAAGTAAA
B
1_ss-A 5′-CCCUCUCCAAACACCGGCAG-3′biotin
1_ss-m6A 5′-CCCUCUCCAA(m6A)CACCGGCAG-3′biotin
2_ss-A 5′-CCGGCAGAAAACGGAGAGUG-3′biotin
2_ss-m6A 5′-CCGGCAGAAA(m6A)CGGAGAGUG-3′biotin
3_ss-A 5′-UUGGAAAGAGACCAGCACCG-3′biotin
3_ss-m6A 5′-UUGGAAAGAG(m6A)CCAGCACCG-3′biotin
4_ss-A 5′-UUAUUUUUGAACCCCUGUGU-3′biotin
4_ss-m6A 5′-UUAUUUUUGA(m6A)CCCCUGUGU-3′biotin
C
YTHDF1
YTHDF2
YTHDF3
YTHDC2
IGF2BP2
IGF2BP3
HNRNPA2B1
HNRNPC
A
m6A
iBAQ
D
SKNO-1
Kasumi-1
YTHDF2
IGFBP2
β-actin
siNS
siYTHDF2
E
SKNO-1
Kasumi-1
YTHDF3
IGFBP2
β-actin
siNS
siYTHDF3

Fig. S6. (related to Fig. 6) Luciferase reporter construction and identification of specific m^{6}A readers targeting the 3′-UTR of *IGFBP2* mRNA. (A) Construction of luciferase reporter vectors. Synthesized wildtype (wt) or mutant (mut) 3′ coding sequences of *IGFBP2* were inserted into the XhoI and NotI site of the psiCHECK2 luciferase reporter. Putative m^{6}A consensus motifs are shown in bold, whereas mutation sites (A to T mutation) are shown in red. (B and C) Identification of m^{6}A specific binding proteins on 3′-UTR of *IGFBP2* by RNA pull-down using 4 pairs of single-stranded RNA (ssRNA) baits containing the 4 m^{6}A consensus motif on the 3′-UTR sequence of *IGFBP2* respectively, with methylated (green) or unmethylated (red) adenosine (B). The iBAQ value of previously reported m^{6}A readers (including YTHDFs, YTHDCs, IG2FBPs and hnRNPs) enriched by the 4 pairs of ssRNA probes detected by mass spectrometry analysis are shown (C). The YTHDC1, YTHDC3, and IGF2BP1 proteins that could not be enriched by all 4 pairs of ssRNA probes are not shown in the Figure (see Table S4). (D and E) Western blot analysis of the expression of IGFBP2 with or without silencing of YTHDF2 (D) or YTHDF3 (E) by siRNA in SKNO-1 or Kasumi-1 cells. siNS, scramble siRNA.

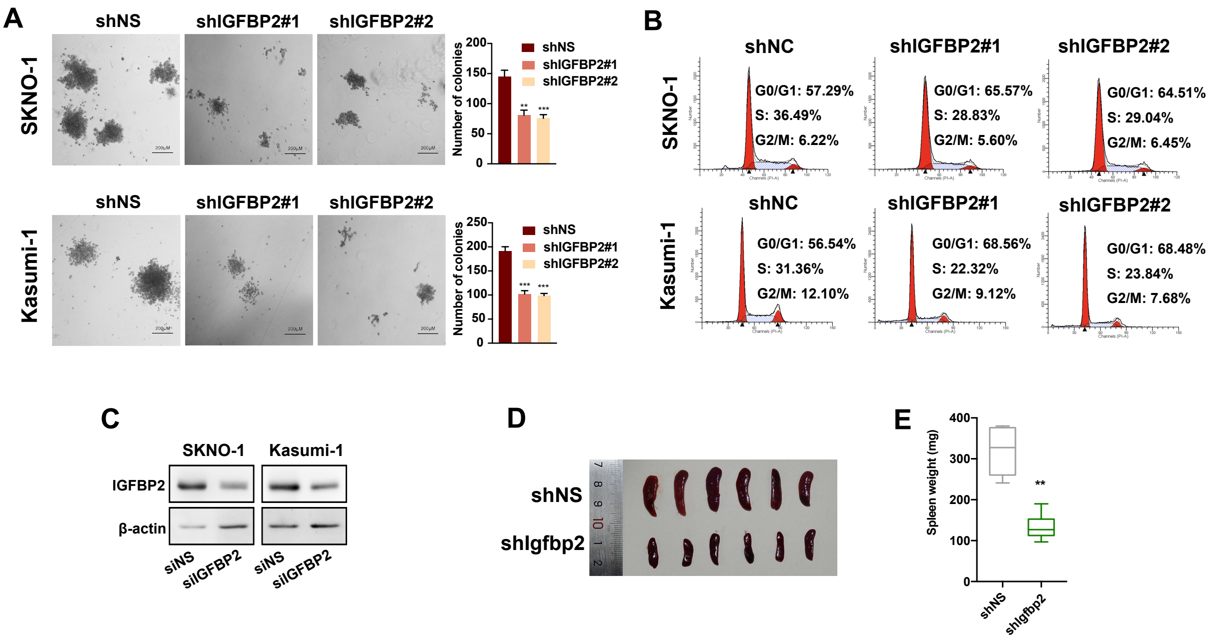

Fig. S7. (related to Fig. 7) Functional role of IGFBP2 in t(8;21) AML. (A and B) Effects of *IGFBP2* knockdown on colony-forming capacity (A) and cell cycle (B) in SKNO-1 and Kasumi-1 cells. (C) Western blot analysis of silencing *IGFBP2* by siRNA in SKNO-1 and Kasumi-1 cells. siNS, scramble siRNA. (D and E) External views (D) and weight (E) of the spleens from AML1-ETO9a-driven AML mice with or without *Igfbp2* knockdown (n = 6 for each group) 7 weeks after transplantation.

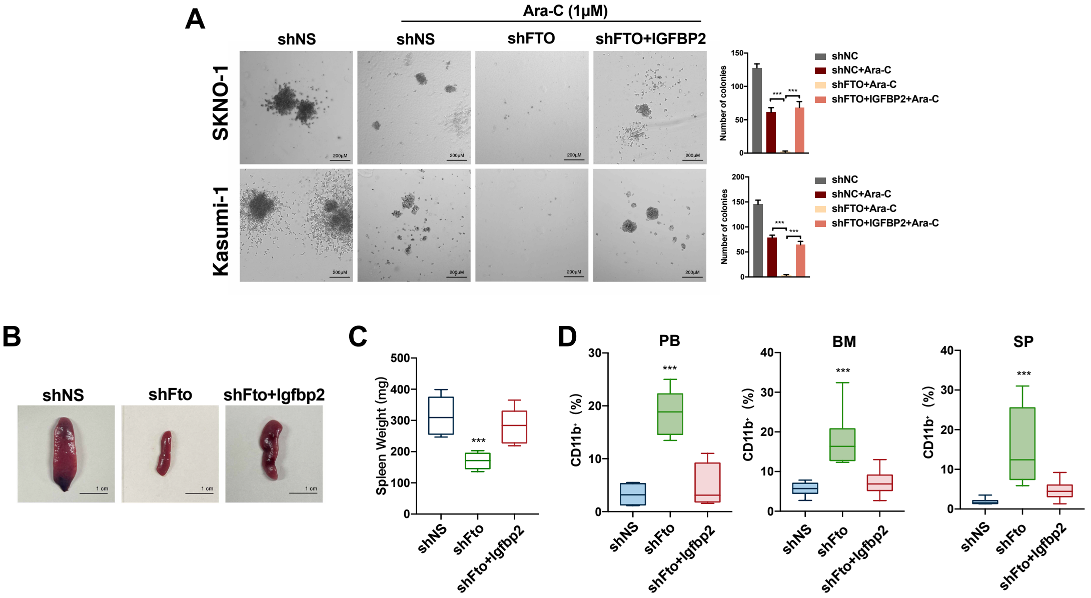

Fig. S8. (related to Fig. 8) FTO regulates leukemogenesis and sensitivity of t(8;21) AML cells to Ara-C through IGFBP2. (A) Effects of *FTO* knockdown with *IGFBP2* overexpression after Ara-C treatment on colony-forming capacity of SKNO-1 and Kasumi-1 cells. (B–D) External views (B), weight of the spleens (C) and flow cytometric analysis of $CD11b^+$ AML cells in PB, BM, and SP (D) of AML1-ETO9a-driven AML mice in Fig. 8E–G.
