# Supplementary material for: A novel AML1-ETO/FTO positive feedback loop promotes leukemogenesis and Ara-C resistance via stabilizing IGFBP2 in t(8;21) acute myeloid leukemia
Source: Exp Hematol Oncol. 2024 Jan 24;13:9. doi: 10.1186/s40164-024-00480-z (PMC10807068; doi:10.1186/s40164-024-00480-z)
Supplement: Supplementary file 2 — Additional file 2: Table S1. The clinical characteristics, morphological and genetic features of patients with de novo t(8;21) AML at diagnosis (n = 26) and relapse (n = 1). Table S2. List of 18 genes that show significant decrease abundance in m6A peak and significant change in the levels of corresponding mRNA transcript between FTO-overexpressing and control Kasumi-1 cells, and are also significantly positively or negatively correlated with FTO in expression in TCGA AML database. Table S3. (Related to Methods) List of oligonucleotides. [file 40164_2024_480_MOESM2_ESM.pdf]

Table S1. The clinical characteristics, morphological and genetic features of patients with de novo t(8;21) AML at diagnosis (n = 26) and relapse (n = 1).

**Pt #**	**Age**	**Sex**	**Karyotype**	**Genotype**	**Disease status of sample**	**Primary refractory AML**
1	29	male	46,XY,inv(9)(p12q13)[2]/46,XY, t(8;21;17)(q22;q22;q22), inv(9)(p12q13)[24]	GATA2/SETD2 mut	at diagnosis	no
2	25	male	45,X,-Y,t(8;21)(q22;q22)[2]/ 46,XY,t(8;21)[1]	None	at diagnosis	Yes
3	13	male	46,XY,t(8;21),del(9)(q13q22)[4]/45,-Y[5]/46, XY[1]	IDH1/NRAS/FAT1/GSTM1/MEF2B/NF1 mut	at diagnosis	No
4	47	female	45, X, -X, t(8;21)(q22;q22)[20]	c-KIT mut	at diagnosis	No
5	46	female	46,XX,t(8;21)(q22;q22)[3]/45,X,-X,t(8;21)(q22;q22)[6]/45,X,-X,t(8;21)(q22;q22),del(9)(q22)[11]	c-KIT/ ASXL1/ PHF6/NOTCH1/ TPMT mut	at diagnosis	No
6	45	male	45,X,-Y,t(8;21)[18]/46,XY[2]	c-KIT mut	at diagnosis	No
7	28	male	45,X,-Y,t(8;21)[19]/46,XY[1]	None	at diagnosis	No
8	27	male	45, -Y, t(8;21) (q22;q22) [4]	FLT3-TKD/c-KIT/NOTCH2/FAT1/IL7R mut	at diagnosis	No
9	60	female	46,XX,t(8;21)(q22;q22)[16]/46,XX,t(8;21)(q22;q22),del(9)(q22)[5]	JAK2 mut	at diagnosis	No
10	16	female	46, XX, t(8;21)(q22;q22)[20]	c-KIT/ NRAS mut	at diagnosis	No
11	45	female	46,XX,t(8;21)[20]	-	at diagnosis	No
12	55	male	45, X, -Y, t(8;21)(q22;q22)[20]	c-KIT mut	at diagnosis	No
13	40	male	46,XY,t(8;21)(q22;q22)[14]/45,X,-Y, t(8;21)(q22;q22)[1]/47,XY,t(8;21)(q22;q22), +21[1]/46, XY[4]	KDM6A/PTEN mut	at diagnosis	Yes
14	26	male	46,XY,t(8;21)(q22;q22)[3]/46,idem, add(2)(q37)[1]/45,idem,-Y[13]/45,idem,-Y, add(2)(q37),-13[2]/44,idem,-Y,add(2)(q37),-18[1]/44, idem, -Y, add(2)(q37), -20[1]/43, idem, -Y, add(2)(q37), -5, -6[1]/40, idem, -Y, add(2)(q37), -5, -5, -7, -19, -21[1]	FLT3-ITD mut	at diagnosis	No
15	43	female	45, X, -X, t(8;21)(q22;q22)[20]	c-KIT/NRAS mut	at diagnosis	No
16	16	male	46,XY,t(8;21)(q22;q22)[20]	None	at diagnosis	yes
17	26	male	46,XY,t(8;21)(q22;q22)[20]	None	at diagnosis	No
18	29	male	46,XY,t(8;21)[19]/47,sl,+2[1]	None	at diagnosis	No
19	37	male	45, X, -Y, t(8;21)(q22;q22)[20]	RAD21/DDX41 mut	at diagnosis	No
20	64	male	45,X,-Y,t(8;21)[7]/44,idem,-5[2]/45,idem,del(5)(q13q31)[4]/45,idem,?der(9)t(9;?)(p12;?)[5]/45,idem,del(5),?der(9)t(9;?)[5]	None	at diagnosis	yes

21	55	female	45,XX,-5,?del(7)(q22q32),-8,-15,-20, ?der(21)t(8;21)(q22;22), +r,+mar1,+mar2[25]/44, XX,-5,?del(7)(q22q32),?add(14)(q34), -20[1]	None	at diagnosis	No
22	53	male	46,XY,t(8;21)(q22;q22)[16]/45,X,-Y, t(8;21)(q22;q22)[4]	CBL, c-KIT mut	at diagnosis	No
23	59	male	46,XY,t(8;21)(q22;q22)[22]/46,XY[1]	None	at diagnosis	Yes
24	38	male	45, X, -Y, t(8;21)(q22;q22)[20]	None	at diagnosis	No
25	20	female	46, XX, t(8;21)(q22;q22)[20]	TPMT mut	at diagnosis	No
26	27	female	45,X,-X,t(1;11)(p32;q13)[17]/45,X,-X, t(1;11)(p32;q13), ?(3;20)(q21;q11)[1]/46, XX[8]	c-KIT mut	at diagnosis	No
27	52	male	46， X， -Y, +der(2)del(2)(p11), 6q-, 7p-, t(8;21)(q22;q22)[10]	c-KIT mut	relapse	No

Table S2 (Related to Figure 5.) List of 18 genes that show significant decrease abundance in m^{6}A peak and significant change in the levels of corresponding mRNA transcript between FTO-overexpressing and control Kasumi-1 cells, and are also significantly positively or negatively correlated with FTO in expression in TCGA AML database[a].

Gene	Change Pattern[b]	m^{6}A abundance changes						mRNA level changes		Correlation between FTO and the given gene in TCGA AML database	
		Peak 1			Peak 2[c]						
		Location	Fold change	p-value	Location	Fold change	P-value	Fold change	P value	Pearson correlation	p-value
ZC3H11A	Hypo-up	3′-UTR	0.78	6.3E-51	3′-UTR	0.83	7.9E-42	3.96	1.1 E-06	0.245	0.001183
IGFBP2	Hypo-up	3′-UTR	0.72	0.001148				2.22	2.2E-12	0.313	2.7E-05
BTBD3	Hypo-up	5′-UTR	0.58	0.009333				2.20	0.01573	0.394	7.8E-08
FTX	Hypo-up	Exon	0.73	1.07E-05	Exon	0.74	1.8E-05	1.84	0.001338	0.283	0.000161
PAX6	Hypo-up	3′-UTR	0.38	0.000398	3′-UTR	0.32	0.001096	1.73	0.02499	0.383	2.0E-07
VKORC1L1	Hypo-up	3′-UTR	0.79	8.5E-08				1.73	0.009957	0.432	2.9E-09
GCC2	Hypo-up	3′-UTR	0.78	2E-12				1.60	0.00065	0.289	0.000116
CEP57	Hypo-up	3′-UTR	0.80	3.9E-14				1.60	2.2E-05	0.327	1.2E-05
TERF2	Hypo-up	3′-UTR	0.72	0.024547				1.54	0.014335	0.228	0.002580
NOA1	Hypo-up	3′-UTR	0.78	9.8E-07				1.52	0.000151	0.313	2.8E-05
YAF2	Hypo-up	3′-UTR	0.83	0.046774				1.50	0.00486	0.237	0.001715
FBXW5	Hypo-down	Exon	0.64	9.5E-05				0.77	0.048858	-0.365	8.1E-07
SERF2	Hypo-down	3′-UTR	0.73	0.046774				0.75	0.035821	-0.310	3.3E-05
SETD7	Hypo-down	3′-UTR	0.49	0.013183				0.70	0.025936	-0.227	0.002705
RAB3A	Hypo-down	3′-UTR	0.67	0.00955				0.66	0.000889	-0.184	0.015630
TCEA3	Hypo-down	3′-UTR	0.77	2.6E-06				0.65	0.041626	-0.177	0.019599
TSPAN14	Hypo-down	3′-UTR	0.77	1.9E-09				0.51	0.011563	-0.478	3.0E-11

GGT1	Hypo-down	3′-UTR	0.66	0.005129	3′-UTR	0.64	0.005754	0.46	0.021192	-0.473	5.1E-11

[a]3′-UTR, 3′ untranslated region; 5′-UTR, 5′ untranslated region; r, correlation coefficient.

[b]Hypo-down and Hypo-up refer to the gene has a significant decrease level in at least one m^{6}A peak ($p < 0.05$; fold change ≤ 0.83) and a significant decrease and increase ($p < 0.05$; fold change ≥ 1.50 or ≤ 0.83) in the overall abundance of the mRNA transcript, respectively, in FTO-overexpressing Kasumi-1 cells compared to the control Kasumi-1 cells;

[c]Four genes have two m^{6}A peaks showing a significant decrease abundance between the FTO-overexpressing and control Kasumi-1 cells.

Table S3 (Related to Methods.) List of oligonucleotides.

Name	Sequence	Note
GAPDH-qPCR-F	CGGATTTGGTCGTATTGGG	qPCR of human genes
GAPDH-qPCR-R	CTGGAAGATGGTGATGGGATT	
FTO-qPCR-F	TGGGTTCATCCTACAACGG	
FTO-qPCR-R	CCTCTTCAGGGCCTTCAC	
AML1-ETO-qPCR-F	ATGACCTCAGGTTTGTCGGTCG	
AML1-ETO-qPCR-R	TGAACTGGTTCTTGGAGCTCCT	
SPI1-qPCR-F	AGTTCCTGTTGGACCTGCTC	
SPI1-qPCR-R	AACTGGAAGGTGCCCTTGTC	
IGFBP2-qPCR-F	GCCCTCTGGAGCACCTCTACT	
IGFBP2-qPCR-R	CATCTTGCACTGTTTGAGGTTGTAC	
YTHDF2-qPCR-F	GGCAGCACTGAAGTTGGG	
YTHDF2-qPCR-R	CTATTGGAAGCCACGATGTTA	
SPI1-Target-F	CTCAGTCCCAGCTTCCTCTG	ChIP-qPCR
SPI1-Target-R	CCAACCCGTTTGCATAAATC	
SPI1-NC-F	CCCCCAGAAAAGATCAGGA	
SPI1-NC-R	ATCCATGCATCTTCCCAATG	
FTO-F1-F	GGCAGAAGAATCGCTTGAAC	
FTO-F1-R	TGTGTGTGACAGGTTCTTGCT	
FTO-F2-F	CCCCGCCTTTATGAAGCTA	
FTO-F2-R	TTGCCCTGGATTAGGAGTTG	
FTO-F3-F	CCTGAGGATGTGGAGGTGTC	
FTO-F3-R	GGTCCTAGGGTCCGCTCT	
FTO-F4-F	ACATGGCAGGCTCCCGTA	
FTO-F4-R	AATTTCCCAGGTCCGACAG	
FTO-F5-F	CTGGGAAATTCTCCTGTGCT	
FTO-F5-R	GTTCTCTCAGCGTCCTGCTC	
FTO-NC-F	GGTTTTCACAGTGGCCATTTA	
FTO-NC-R	AAAATCCCCCTCTAGCCTGA	
AML1-ETO-m^{6}A-F	ATGACCTCAGGTTTGTCGGTCG	m^{6}A-qPCR
AML1-ETO-m^{6}A-R	TGAACTGGTTCTTGGAGCTCCT	
IGFBP2-m^{6}A-F	GCCCTCTGGAGCACCTCTACT	
IGFBP2-m^{6}A -R	CATCTTGCACTGTTTGAGGTTGTAC	
PAX6-m^{6}A-F	TGGGCAGGTATTACGAGACTG	
PAX6-m^{6}A-R	ACTCCCGCTTATACTGGGCTA	
Cep57-m^{6}A-F	AAGCCTACACTTGCCTATCCAG	
Cep57-m^{6}A-R	TTCCAAGCGTCGAATCTTATC	
TERF2-m^{6}A-F	CTGAGCTCACACCACTGGAA	
TERF2-m^{6}A-R	GCATCTTCTGCTGGAAGGTC	
FBXW5-m^{6}A-F	CTGGTACGAGGAGTTCCAGC	
FBXW5-m^{6}A-R	TGCTCCAGATCTTCACAGTGC	
Rab3A-m^{6}A-F	GAGTCCTCGGATCAGAACTTCG	

Rab3A-m^{6}A-R	TGTCGTTGCGATAGATGGTCT	
TCEA3-m^{6}A-F	TGTCCTTGGCCAAAGTCC	
TCEA3-m^{6}A-R	GGAGAAAGGCCTGCTTCTG	
DNMT3A-m^{6}A-F	GGAGGACCGAAAGGACGGA	
DNMT3A-m^{6}A-R	CCCCATTGGGTAATAGCTCTGAG	
DNMT1-m^{6}A-F	GTGGGGGACTGTGTCTCTGT	
DNMT1-m^{6}A-R	TGAAAGCTGCATGTCCTCAC	
AML1-ETO-RIP-F	ATGACCTCAGGTTTGTCGGTCG	RIP
AML1-ETO-RIP-R	TGAACTGGTTCTTGGAGCTCCT	
IGFBP2-RIP-F	GCCCTCTGGAGCACCTCTACT	
IGFBP2-RIP-R	CATCTTGCACTGTTTGAGGTTGTAC	
FTO_P1_forward	CGGGGTACCGGAGAAACATGGCAGGCTCCCGT	FTO promoter amplification for Dual-luciferase reporter assays
FTO_P2_forward	CGGGGTACCGTTTGCTCGCGGGGTAGCGGACT	
FTO_P3_forward	CGGGGTACCGTTTTTTCTACTCAGAGGGAGAA	
FTO_P4_forward	CGGGGTACCGCGGGGTCCAGGGCGAGGGA	
FTO_P1-4_reverse	CCCAAGCTT GCTGCCACTAAAGCCGCCT	
